# Comparative Genomics Assisted Functional Characterization of *Rahnella aceris* ZF458 as a Novel Plant Growth Promoting Rhizobacterium

**DOI:** 10.3389/fmicb.2022.850084

**Published:** 2022-04-04

**Authors:** Shuai Xu, Yurong Zhao, Yue Peng, Yanxia Shi, Xuewen Xie, Ali Chai, Baoju Li, Lei Li

**Affiliations:** Institute of Vegetables and Flowers, Chinese Academy of Agricultural Sciences, Beijing, China

**Keywords:** *Rahnella aceris* ZF458, comparative genomic analysis, biological control, plant growth promotion, environmental adaptation

## Abstract

Many *Rahnella* strains have been widely described as plant growth-promoting rhizobacteria with the potential to benefit plant growth and protect plants from pathogens. *R. aceris* ZF458 is a beneficial plant bacterium isolated from swamp soil with the potential for biocontrol. Strain ZF458 has shown broad-spectrum antagonistic activities against a variety of plant pathogens and exhibited a dramatic effect on controlling *Agrobacterium tumefaciens* in sunflowers. The *R. aceris* ZF458 genome sequence contained a 4,861,340-bp circular chromosome and two plasmids, with an average G + C content of 52.20%. Phylogenetic analysis demonstrated that *R. aceris* ZF458 was closely related to *R. aceris* SAP-19. Genome annotation and comparative genomics identified the conservation and specificity of large numbers of genes associated with nitrogen fixation, plant growth hormone production, organic acid biosynthesis and pyrroloquinoline quinone production that specific to benefiting plants in strain ZF458. In addition, numerous conserved genes associated with environmental adaption, including the bacterial secretion system, selenium metabolism, two-component system, flagella biosynthesis, chemotaxis, and acid resistance, were also identified in the ZF458 genome. Overall, this was the first study to systematically analyze the genes linked with plant growth promotion and environmental adaption in *R. aceris*. The aim of this study was to derive genomic information that would provide an in-depth insight of the mechanisms of plant growth-promoting rhizobacteria, and could be further exploited to improve the application of *R. aceris* ZF458 in the agriculture field.

## Introduction

*Rahnella* was a Gram-negative, rod-shaped, facultatively anaerobic bacterium belonging to the family *Yersiniaceae* (Adeolu et al., 2016), and was widely distributed in a variety of environments, including soil, phyllosphere, water, seeds, food, some clinical samples, and even in American mastodon remains (Berge et al., 1991; Rhodes et al., 1998). *Rahnella* was first proposed in 1979, and normally consisted of six species including *R. aquatilis* (which was described as the type species of *Rahnella*), *R. variigena*, *R. inusitata*, *R. bruchi*, *R. woolbedingensis*, and *R. victoriana* by multilocus sequence analysis (Brady et al., 2014). Recently, a new species named *R. aceris* was proposed in 2020, which shared a high similarity with the *R. aquatilis* (Lee et al., 2020). To date, only one *R. aceris* strain SAP-19 has been reported for general physiological and biochemical characteristics, shotgun genome information and phylogenetic analyses (Lee et al., 2020). Since there have been very few studies on *R. aceris*, its genetic background, taxonomic classification and potential biological function were still unclear.

Decades of research have proven that *Rahnella* spp. could be applied to preventing and controlling plant disease or promoting plant growth for their beneficial properties. Many *Rahnella* strains have been described to protect plants from a wide range of phytopathogenic organisms in different ways. *R. aquatilis* JZ-GX1 secreted volatile organic compounds (VOCs) that destroyed the mycelial growth of *Colletotrichum gloeosporioides*, and thus restrained the infection and expansion of anthracnose disease in leaves (Kong et al., 2020a). *R. aquatilis* Ra36 could exploit fungal chemotropism to efficiently colonize the roots of host plants, resulting in efficient protection from vascular wilt disease in tomatoes by interfering with the alkaline-triggered infection of *Fusarium oxysporum via* gluconic acid secretion (Palmieri et al., 2020). *R. aquatilis* HX2 was proven to decrease the disease incidence of crown gall disease caused by *Agrobacterium vitis* in grapevines by producing antibacterial substances (Chen et al., 2009). Besides, *R. aquatilis* Ra39 exhibited a significant effect on controlling *Erwinia amylovora* in apple, and the control efficiency was close to the efficacy of streptomycin when the strain was mixed with the Acibenzolar-S-methyl (ASM) (Abo-Elyousr et al., 2010). Application of *R. aquatilis* Ra to bean plants increased the content of phenolic compounds and the activity of peroxidase (PO) enzyme than untreated plants, and resulted in a marked disease suppression against *Xanthomonas axonopodis* pv. *phaseoli* under greenhouse and field conditions (Sallam, 2011). *R. aquatilis* 17 and 55 exhibited a marked siderophore production and a significant effect on controlling the bacterial spot of cucumber (el-Hendawy et al., 2003). In addition, many *Rahnella* strains have been demonstrated to benefit plant growth through various mechanisms, such as nitrogen fixation, phytohormone production, phosphate solubilization, biosynthesis of organic acids and pyrroloquinoline quinone. For example, *R. aquatilis* ZF7 significantly promoted the weight of aboveground parts and the root length in Chinese cabbage due to its high Indole-3-acetic acid (IAA) biosynthetic capacity (Yuan et al., 2020). *R. aquatilis* HX2 exhibited a significant promotion of the growth of corn, due to its ability to solubilize mineral phosphate, produce pyrroloquinoline quinine and IAA (Guo et al., 2009). *R. aquatilis* JZ-GX1 exhibited a significant greening effect on *Cinnamomum camphora* by producing organic acids (Kong et al., 2020b), and was also reported as a phytate-degrading rhizobacteria (PDRB) that could improve the growth of poplar and Masson pine (Li et al., 2013). To date, the majority of studies on plant growth promotion in *Rahnella* spp. focused on *R. aquatilis* strains. Given the close relationship between *R. aceris* and *R. aquatilis*, the *R. aceris* strains may possess similar mechanisms of biocontrol and plant growth promotion.

Previous studies have demonstrated that *Rahnella* strains were widely distributed and adapted to diverse ecological environments, which might be related to their resistance to acids, salts, selenium, antibiotics and heavy metals. For example, *R. aquatilis* HX2 exhibited a tolerance to high salt, strong acids, antibiotics, and stress tolerance (Li et al., 2019, 2021). In addition, *R. aquatilis* HX2 could grow in high selenium concentrations and could reduce selenate and selenite to BioSeNPs (Zhu et al., 2018). *R. aquatilis* JZ-GX1 could stimulate the production of exopolysaccharides, and resulting in high salt tolerance to 0–9% NaCl (Li et al., 2021). *R. aquatilis* ZF7 harbored 24 genes associated with the biosynthesis and metabolism of β-lactamase, and exhibited tolerance to a variety of antibiotics including ampicillin, carbenicillin and vancomycin (Yuan et al., 2020). *Rahnella* sp. SMO-1 was reported to encode extended-spectrum β-lactamase which could efficiently hydrolyze penicillins and cefotaxime, and the strain was also showed resistance to amoxicillin, ticarcillin, cefalotin and cefuroxime (Lartigue et al., 2013). In addition, *Rahnella* sp. JN6 showed a high resistance to Cd, Pb, and Zn and could improve the efficiency of remediation in heavy metal contaminated soils (He et al., 2013). Previous studies have indicated that a range of regulatory mechanisms including secretory system, two-component regulatory system (TCS) and acid-resistance genes played important roles in the environmental adaption of bacteria (Liu and Ochman, 2007). For instance, multiple secretion systems were found in *Yersinia enterocolitica* and were essential for virulence, the biosynthesis of antibiotics and the life activities (Heermann and Fuchs, 2008). *Photorhabdus luminescens* harbored 18 TCSs which had been found to be involved in metabolite utilization and adaptation to various stress factors (Heermann and Fuchs, 2008). Nevertheless, relatively few studies have focused on adapting to environmental variations in *Rahnella* strains. Therefore, to better promote the application prospects of *Rahnella* spp., studying the environmental adaption of *Rahnella* strains, especially at the molecular level was very necessary.

In this study, *R. aceris* ZF458 with broad spectrum of antagonistic activities against many plant pathogens was isolated from swamp soil. Phylogenetic analysis, ANI and DDH analysis were accomplished to definite the taxonomic position of ZF458 and the relationship with *R. aceris* SAP-19 and other *R. aquatilis* strains. The whole genome of ZF458 was sequenced, annotated and compared with the genomes of other typical and widely reported *Rahnella* strains HX2, ZF7, Y9602 and ATCC 33071^T^. Comparative genome analysis was used to reveal various genes involved in biocontrol factors and environmental adaption. These data would provide an in-depth insight into the mechanisms of plant growth promotion, and improve the bio-control application of *R. aceris* ZF458 in the future.

## Materials and Methods

### Strain Isolation, Antagonistic Assays and Biocontrol Assays

Stain ZF458 was isolated from swamp soil in Sichuan, China, in August, 2019, according to a standard 10-fold dilution plating assay as described by previous study (Wang B. et al., 2013). The antagonistic activities of *R. aceris* ZF458 against plant pathogens were performed by plate tests, and the control effect of ZF458 against *Agrobacterium tumefaciens* was tested on sunflowers. The growth curve of strain ZF458 was measured according to the following steps: strain ZF458 was incubated overnight at 28°C, and the suspension was diluted to an OD_600_ = 0.6 with sterile Nutrient Broth (NB) medium. Subsequently, 1 μL of the suspension was added to 999 μL NB culture *via* vortexing. Then, 200 μL of the diluent was dripped to a 96 well plate. The microtiter plates were kept in a stable shaking speed (180 rpm⋅min^–1^), and the OD_600_ of ZF458 culture was measured at 4 h intervals over 40 h. In addition, plate assays were conducted to assess siderophore and phosphatase activities based on reported approaches (Milagres et al., 1999; Luis et al., 2014). If siderophores were produced, the chrome azurol S (CAS) agar would change from blue to orange, while if phosphatases were produced, the Pikovskaya’s (PVK) agar plate would change from white to transparent.

### Growth Conditions, Microscopic Analysis and Genomic DNA Extraction

Strain ZF458 was incubated in NB medium at 28°C with shaking for 24 h. The morphology of strain ZF458 was observed by scanning electron microscope (SEM) Hitachi-S3400N and transmission electron microscope (TEM) Hitachi-7700. The genomic DNA of strain ZF458 was extracted from cultured cells (OD_600_ = 0.8) by TIANamp Bacteria DNA kit (Tiangen Biotech (Beijing) Co., Ltd.).

### Genome Sequencing and Annotation

The whole genome of *R. aceris* ZF458 was sequenced in Allwegene Technologies Corporation, China. The pacific Biosciences (PacBio) RS II platform was used for the whole-genome sequencing of strain ZF458, and a 10-kb SMRT Bell was used for template library construction. The sequence reads were assembled *de novo* using SMRT Link v.5.1.0.^1^ CGView was used to generate the circular genome visualization (Stothard and Wishart, 2005). GeneMarkS (version 4.17) software was used to predict the coding genes of the sequenced genome.^2^ Transfer RNA (tRNA), ribosomal RNA (rRNA) and small nuclear RNAs (snRNAs) were predicted using tRNAscan-SE version 1.3.1 (Lowe and Eddy, 1997), rRNAmmer version 1.2 (Lagesen et al., 2007) and cmsearch version 1.1 (Nawrocki et al., 2009), respectively. Functional gene annotation was carried out through multiple general databases, including Non-Redundant protein databases (NR) (Li et al., 2002), Gene Ontology database (GO) (Ashburner et al., 2000), Kyoto Encyclopedia of Genes and Genomes (KEGG) (Kanehisa et al., 2006), Cluster of Orthologous Groups of proteins (COG) (Galperin et al., 2015), Transporter Classification Database (TCDB) (Saier et al., 2009), Pfam,^3^ Swiss-Prot^4^ (Amos and Rolf, 2000), Carbohydrate-Active enZTmes Database (CAZy) (Cantarel et al., 2009). Furthermore, signal peptides and transmembrane structure were predicted using SignalP 4.1 and TMHMM 2.0c (Petersen et al., 2011). In addition, prophages were predicted by using phiSpy 2.3 (You et al., 2011), and CRISPR (Clustered Regularly Interspaced Short Palindromic Repeat Sequences) was identified *via* CRISPRdigger 1.0 (Ibtissem et al., 2007).

### Phylogenetic Analysis and Comparative Genomic Analysis

Multilocus gene sequence analysis (MLSA) based on the four housekeeping genes (16S rRNA, *gyr*B, *atp*D, and *rpo*B) was used to determine the taxonomic position of *R. aceris* ZF458. The housekeeping gene sequences of diverse strains (Supplementary Table 1) were aligned using MUSCLE and the phylogenetic tree was generated using the maximum likelihood method by MEGA 6.0 (Tamura et al., 2013). Average nucleotide identities (ANI) and *in silico* DNA-DNA hybridization (DDH) analysis were performed using the OrthoANIu algorithm and the Genome-to-Genome Distance Calculator (GGDC), respectively (Meier-Kolthoff et al., 2013). Based on the phylogenetic analysis, four closely related *Rahnella* strains including *R. aquatilis* ZF7 (CP032296.1), *R. aquatilis* HX2 (CP003403.1), *Rahnella* sp. Y9602 (CP002505.1) and *R. aquatilis* ATCC 33071^T^ (CP003244.1) were selected for comparative genomic analysis. Moreover, pairwise alignment of genomes was generated using the Mauve 2.3.1 comparison software, and Venn diagrams were conducted using R package (Richter and Rossello-Mora, 2009).

### Functional Gene Analysis Linked With Plant Growth Promotion and Environmental Adaption

Functional genes beneficial to plants such as IAA production, nitrogen fixation, phosphate solubilization, organic acid and pyrroloquinoline quinone (PQQ) biosynthesis, were searched for in the NCBI database. In addition, functional genes involved in environmental adaption including bacteria secretion system, flagella biosynthesis and chemotaxis, two-component system, quorum sensing, SeNPs production, acid-resistance were detected using the NCBI database. All the functional genes retrieved from the ZF458 genome were compared at the amino acid level with four closely related *Rahnella* strains ZF7, HX2, Y9602 and ATCC 33071^T^ by KEGG database.

## Results

### Antagonistic Activity and Biological Characteristics of Strain ZF458

#### Biocontrol Traits of Strain ZF458

**FIGURE 1:**
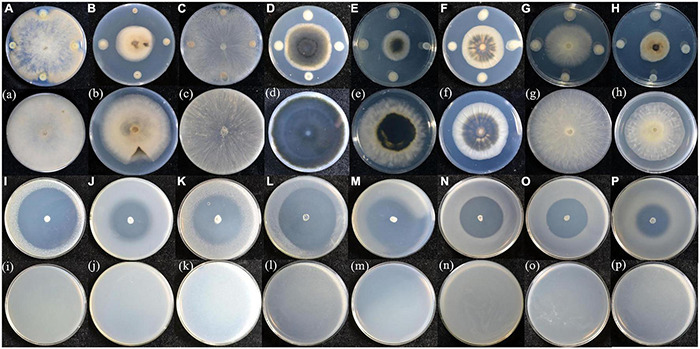
Antagonistic assays of *R. aceris* ZF458 against various plant fungal and bacterial pathogens. **(A,a)** *Botrytis cinerea*, **(B,b)** *Stemphylium solani*, **(C,c)** *Rhizoctonia solani*, **(D,d)** *Corynespora cassiicola*, **(E,e)** *Ascochyta citrulline*, **(F,f)** *Colletotrichum* sp., **(G,g)** *Fusarium oxysporum*, **(H,h)** *Phytophthora capsici*, **(I,i)** *Pectobacterium brasiliense*, **(J,j)** *Pseudomonas amygdali* pv. *lachrymans*, **(K,k)** *Pseudomonas syringae* pv. *tomato*, **(L,l)** *Ralstonia solanacearum*, **(M,m)** *Xanthomonas campestris* pv. *campestris*, **(N,n)** *Clavibacter michiganensis* subsp. *michiganensis*, **(O,o)** *Agrobacterium tumefaciens*, **(P,p)** *Acidovorax citrulli*. The capital letters represent plate containing strain ZF458, the small letters represent control.

Strain ZF458 which was isolated from swamp soil exhibited broad, obvious antagonistic activities against diverse plant-pathogenic fungi and bacteria, including *Stemphylium solani*, *Corynespora cassiicola*, *Ascochyta citrulline*, *Colletotrichum* sp., *Phytophthora capsici*, *Fusarium oxysporum*, *Pectobacterium brasiliense*, *Pseudomonas amygdali* pv. *lachrymans*, *Pseudomonas syringae* pv. *tomato*, *Ralstonia solanacearum*, *Xanthomonas campestris* pv. *campestris*, *Clavibacter michiganensis* subsp. *michiganensis*, *Agrobacterium tumefaciens*, and *Acidovorax citrulli* (Figure 1). Importantly, strain ZF458 presented a dramatic effect in controlling *Agrobacterium tumefaciens* on sunflowers (Supplementary Figure 1), with a control efficiency of 80.20%. Moreover, orange halos were appeared around colonies of strain ZF458 on CAS agar plates, revealing that strain ZF458 produced siderophores (Supplementary Figure 2A). Similarly, transparent halos were observed around ZF458 colonies on PVK agar plates, indicating that strain ZF458 produced phosphatases (Supplementary Figure 2B).

#### Organism Information

Strain ZF458 was determined to be a Gram-negative, facultatively anaerobic bacterium belonging to the *Yersiniaceae* family. The strain produced white to light yellow-colored, circular, translucent, convex with margin colonies and reached 0.1–5.0 mm in a diameter on 1-day culture on NA plate at 28°C (Supplementary Figure 3A). Strain ZF458 displayed shot rod-shaped cells with lengths of 1.5–3.0 μm and diameters of 0.7–1.3 μm (Supplementary Figures 3B,C). The growth curve showed that strain ZF458 was in exponential growth phase between 4–20 h after inoculation, and attained the stationary phase at 20 h of incubation (Supplementary Figure 4A). Furthermore, the pH of strain ZF458 culture declined rapidly from approximately 7.0 to 3.2 within 12 h of incubation, and remained stable after 12 h (Supplementary Figure 4B).

### Genome Structure and Genome Comparison Between *R. aceris* ZF458 and Other Completely Sequenced *Rahnella* Strains

#### General Genomic Features of *R. aceris* ZF458

**FIGURE 2:**
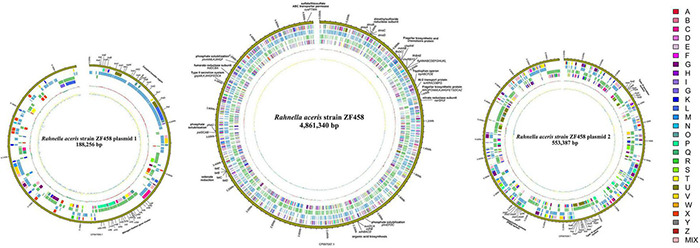
Graphical circular maps of the *R. aceris* ZF458 chromosome and plasmids pPlas1 and pPlas2 genome generated using the CGview server. From outside to center, ring 1 showed the genome sequence position, rings 2 and 5 showed protein-coding genes oriented in the forward (colored by COG categories) and reverse (colored by COG categories) directions, respectively. Rings 3 and 4 showed coding genes in the forward (blue) and reverse (green) directions, respectively. Ring 6 showed the G + C% content and the inner most ring showed the GC skews, where blue indicated positive values and yellow indicated negative values.

The whole genome of *R. aceris* ZF458 was 5.60 Mb with an overall G + C content of 52.20% (Supplementary Table 2). The entire genome comprised a circular 4,861,340-bp chromosome (CP067057.1) and two plasmids with sizes of 188,256-bp (CP067058.1) and 553,387-bp (CP067059.1). The general genome structure and functions of strain ZF458 was represented by graphical circular genome map (Figure 2). In total 5,248 predicted genes were identified in the genome, including 4,988 protein coding genes, 109 RNA genes and 88 pseudogenes. According to KEGG database, a total of 4,954 genes of ZF458 were annotated to 40 different pathways which were associated with metabolism, cellular processes, environmental information processing, genetic information processing and organismal systems. By comparing the Swiss-Prot, Pfam, TCDB, SinalIP and CAZy databases, 3,316 (63.19%), 3,533 (67.32%), 939 (17.89%), 428 (8.16%), and 210 (4.00%) of the ORFs were annotated into different groups, respectively. Besides, the ZF458 genome contained 342 secreted proteins, 10 genomic islands and 7 prophage regions (Supplementary Table 2).

Moreover, a total of 4,237 functional genes of ZF458 were further annotated using COG database. Most of the genes were related to amino acid transport and metabolism (10.57%), followed by carbohydrate transport and metabolism (9.86%), transcription (8.68%), inorganic ion transport and metabolism (6.66%), cell wall/membrane biogenesis (6.21%) and translation, ribosomal structure and biogenesis (5.17%) (Table 1). However, 83 genes were not annotated by COG database, whose specific functions need to be further verified.

**TABLE 1:** Number of genes associated with general COG functional categories.

Code	Value	% age	Description
J	258	5.17	Translation, ribosomal structure and biogenesis
A	1	0.02	RNA processing and modification
K	433	8.68	Transcription
L	134	2.69	Replication, recombination and repair
D	47	0.94	Cell cycle control, Cell division, chromosome partitioning
V	125	2.51	Defense mechanisms
T	243	4.87	Signal transduction mechanisms
M	310	6.21	Cell wall/membrane biogenesis
N	115	2.31	Cell motility
W	43	0.86	Extracellular structures
U	117	2.35	Intracellular trafficking and secretion
O	170	3.41	Posttranslational modification, protein turnover, chaperones
C	235	4.71	Energy production and conversion
G	492	9.86	Carbohydrate transport and metabolism
E	527	10.57	Amino acid transport and metabolism
F	110	2.21	Nucleotide transport and metabolism
H	208	4.17	Coenzyme transport and metabolism
I	162	3.25	Lipid transport and metabolism
P	332	6.66	Inorganic ion transport and metabolism
Q	116	2.33	Secondary metabolites biosynthesis, transport and catabolism
R	395	7.92	General function prediction only
S	232	4.65	Function unknown
X	100	2.00	Mobilome; prophages, transposons
−	83	1.66	Not in COGs

*The total % age is based on the total number of protein coding genes in the annotated genome.*

#### Phylogenetic Tree

To understand the genetic relationships of *R. aceris* ZF458 with other related strains especially the *Rahnella* strains, a phylogenetic tree was established based on 16S rRNA and three other housekeeping genes (*gyr*B, *atp*D and *rpo*B). As expected, seventeen *Rahnella* strains were clustered into one major clade, and three *Rouxiella* strains, two *Yersinia* strains, two *Serratia* strains were in other clades (Supplementary Figure 5). The phylogenetic analysis indicated that 17 *Rahnella* strains including ZF458 were in seven *Rahnella* MLSA groups. Based on the observed genetic distance relationships, strain ZF458 was closely clustered together with *R. aceris* SAP-19, *R. aquatilis* ZF7, *R. aquatilis* HX2, *Rahnella* sp. Y9602 and the type strain *R. aquatilis* ATCC 33071^T^, successively followed by *R. victoriana* strains, *R. variigena* strains, *R. bruchi* strains, *R. woolbedingensis* strains, and *R. inusitata* strains.

#### Average Nucleotide Identities and DNA-DNA Hybridization Analysis

Average nucleotide identities and DDH were widely used to certify the genetic distance of bacteria at the genomic level, and strains revealing ANI values ≥96% and DDH values ≥70% were typically considered as the same species (Zhang et al., 2016). In the study, ANI and DDH calculations among *Rahnella* strains were performed. The results showed that the ANI values between ZF458 and SAP-19, ZF7, HX2, Y9602, KM05, KM12, MEM40 were both >98%, and the DDH values among them were both >80% (Supplementary Figure 6). These findings demonstrated that the eight *R. aceris* strains or *R. aquatilis* strains (ZF458, SAP-19, ZF7, HX2, Y9602, KM05, KM12, and MEM40) were closely clustered with one another and occupied a close genetic distance relationship. However, the ANI and DDH values between strains ZF458 and ATCC 33071^T^ were 92.75 and 49.4% respectively, although the two strains were clustered closely in the phylogenetic tree (Supplementary Figure 5). Smaller ANI and DDH values were calculated when other *Rahnella* strains (except the *R. aceris* and *R. aquatilis* species) were used as reference genomes.

#### Genomic Features of Different *Rahnella* Strains

In the study, four *Rahnella* strains that have completed whole genome sequencing including *R. aquatilis* ZF7, *R. aquatilis* HX2, *Rahnella* sp. Y9602, and *R. aquatilis* ATCC 33071^T^ were selected for comparative genomic analysis (Table 2). In comparison, the entire genome size of the five *Rahnella* strains ranged from 5.45 to 5.66 Mb, the G + C content ranged from 52.08 to 52.36%, and the predicted coding genes ranged from 4,804 to 5,065. The results illustrated that the genome size of strain ZF458 was larger than that of ZF7 and ATCC 33071^T^, but smaller than that of HX2. Furthermore, the genomes of strains ZF458 and Y9602 contained one circular chromosome and two plasmids, while HX2 and ATCC 33071^T^ contained one circular chromosome and three plasmids, and the sequenced strain ZF7 harbored a circular chromosome and only one plasmid (Table 2).

**TABLE 2:** Genomic features of *Rahnella aceris* ZF458 and other *Rahnella* spp.

Features	*Rahnella aceris* ZF458	*Rahnella aquatilis* ZF7	*Rahnella aquatilis* HX2	*Rahnella* sp. Y9602	*Rahnella aquatilis* ATCC 33071
Size (Mb)	5.60	5.54	5,66	5.61	5.45
G + C content (%)	52.20	52.36	52.15	52.18	52.08
Replicons	One chromosome Two plasmids	One chromosome One plasmid	One chromosome Three plasmids	One chromosome Two plasmids	One chromosome Three plasmids
Total genes	5,185	5,115	5,208	5,217	4,989
Predicted no. of CDS	4,988	4,936	4,991	5,065	4,804
Ribosomal RNA	22	22	22	22	22
Transfer RNA	78	77	76	76	76
Other RNA	9	10	8	8	10
Pseudogene	88	60	111	46	77
GenBank sequence	CP067057.1	CP067057.1	CP003403.1	CP002505.1	CP003244.1

#### Comparison of *R. aceris* ZF458 With the Four Typical *Rahnella* Strains

**FIGURE 3:**
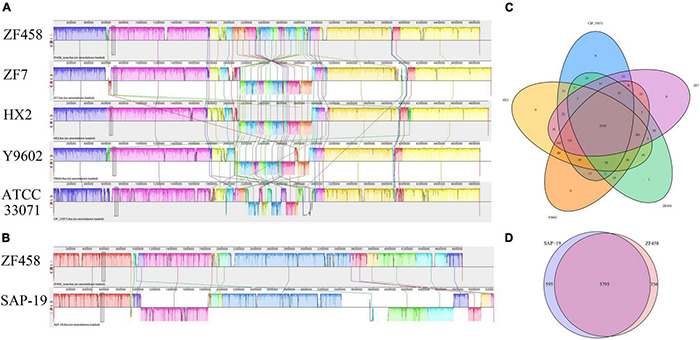
Global alignment of the genome sequence of completely sequenced *R. aceris* ZF458 against five other *Rahnella* genome sequences. **(A)** Mauve progressive alignment of the ZF458, ZF7, HX2, Y9602, and ATCC 33071 genomes. **(B)** Mauve progressive alignment of the ZF458 and SAP-19 genomes. ZF458 genome was used as the reference genome. Boxes with the same color indicate syntenic regions. Boxes below the horizontal strain line indicate inverted regions. Rearrangements are indicated with the colored lines. The scale is in nucleotides. **(C)** Venn diagram showing the numbers of shared and unique Clusters of Orthologous Genes among ZF458, ZF7, HX2, Y9602, and ATCC 33071 genomes. **(D)** Venn diagram showing the numbers of shared and unique Clusters of Orthologous Genes between the ZF458 and SAP-19 genomes.

In addition, the entire genome of ZF458, SAP-19, ZF7, HX2, Y9602 and ATCC 33071^T^ were compared using Mauve to assess the homology among these *Rahnella* strains. The results showed that more local collinear blocks (LCB) inversion was obviously observed between strains ZF458 and ATCC 33071^T^ than that among strains ZF458, ZF7, HX2 and Y9602 (Figure 3A). This indicated that the ZF458 genome was highly syntenic with *R. aquatilis* ZF7, *R. aquatilis* HX2 and *Rahnella* sp. Y9602. At the species level, less local collinear blocks inversion was found between strains ZF458 and SAP-19, but compared with the ZF458 genome, large local collinear blocks (LCB) translocation occurred in the genome of SAP-19 (Figure 3B).

As shown in Figure 3C, 3,530 conserved genes were shared by strains ZF458, ZF7, HX2, Y9602 and ATCC 33071^T^. ZF458 shared 3,893 genes with ZF7, 3,884 genes with HX2, and 3,928 genes with counterparts in the Y9602 genome, while only 3,647 orthologous genes existed in strains ZF458 and ATCC 33071. Interestingly, no unique genes were found in the genome of *R. aquatilis* strains ZF7, HX2, Y9602 and ATCC 33071^T^, while one unique gene was found in ZF458 genome (Figure 3C). In addition, 3,795 orthologous genes were found between the ZF458 and SAP-19 genomes, and 356 unique genes existed in the ZF458 genome were not retrieved in the genome of SAP-19 (Figure 3D).

### Synergistic Interactions With the Host Plant: Benefiting Plants

#### Nitrogen Fixation and Metabolism

To compare the key genes associated with the plant growth promotion in *R. aceris* ZF458, four strains including ZF7, HX2, Y9602 and ATCC 33071^T^ were selected. The results showed that a relatively whole *nif* gene cluster with 21 genes related to nitrogen fixation was retrieved in the genomes of strains ZF458, HX2 and ATCC 33071^T^ (Table 3). All the genes were assigned to Mo nitrogenase structural genes, FeMo-co synthesis, maturation of nitrogenase, electron transport, regulation of *nif* gene expression and three unknown functional genes, and shared high similarities among the three strains. However, only one gene *nif*J encoding pyruvate ferredoxin/flavodoxin oxidoreductase was present in the ZF7 genome, and indeed, the *nif* gene cluster which existed in strains ZF458, HX2 and ATCC 33071^T^ was found in the plasmid genome, except the gene *nif*J, which was retrieved from the chromosome genome. In addition, 7 highly homologous nitrogen regulation related genes (*gln*ABDGO, *ntr*B, and *nac*) were found in the genomes of *R. aceris* ZF458 and four other *Rahnella* strains (Table 3).

**TABLE 3:** Homolog analysis of nitrogen fixing gene cluster in *R. aceris* ZF458 and other *Rahnella* strains.

Strain		*Rahnella aceris* ZF458		*R. aquatilis* ZF7		*R. aquatilis* HX2		*Rahnella* sp. Y9602		*R. aquatilis* ATCC 33071	
Genes	Product definition	Locus Tag	Protein ID	Protein ID	Homology (%)	Protein ID	Homology (%)	Protein ID	Homology (%)	Protein ID	Homology (%)
**Mo nitrogenase structural genes**											
*nifH*	Nitrogenase iron protein NifH	JHW33_RS22855	WP_014333921.1	NA	NA	WP_014333921.1	100	NA	NA	WP_014333921.1	100
*nifD*	Nitrogenase molybdenum-iron protein alpha chain	JHW33_RS22850	WP_200227561.1	NA	NA	WP_014683022.1	99	NA	NA	WP_014333920.1	99
*nifK*	nitrogenase molybdenum-iron protein subunit beta	JHW33_RS22845	WP_014683023.1	NA	NA	WP_014683023.1	100	NA	NA	WP_014333919.1	99
**FeMo-co Synthesis**											
*nifY*	Nitrogen fixation protein NifY	JHW33_RS22835	WP_014683024.1	NA	NA	WP_014683024.1	100	NA	NA	WP_014333917.1	97
*nifE*	Nitrogenase molybdenum-cofactor synthesis protein NifE	JHW33_RS22830	WP_014683025.1	NA	NA	WP_014683025.1	100	NA	NA	WP_014333916.1	99
*nifN*	Nitrogenase molybdenum-cofactor biosynthesis protein NifN	JHW33_RS22825	WP_014683026.1	NA	NA	WP_014683026.1	100	NA	NA	WP_014333915.1	99
*nifX*	nitrogen fixation protein NifX	JHW33_RS22820	WP_014683027.1	NA	NA	WP_014683027.1	100	NA	NA	WP_014333914.1	98
*nifU*	Fe-S cluster assembly protein NifU	JHW33_RS22810	WP_014683029.1	NA	NA	WP_014683029.1	100	NA	NA	WP_014333912.1	97
*nifS*	Cysteine desulfurase	JHW33_RS22805	WP_200227559.1	NA	NA	WP_014683030.1	99	NA	NA	WP_014333911.1	99
*nifV*	Homocitrate synthase NifV	JHW33_RS22800	WP_014683031.1	NA	NA	WP_014683031.1	100	NA	NA	WP_014333910.1	98
*nifQ*	Nitrogen fixation protein NifQ	JHW33_RS22765	WP_014683038.1	NA	NA	WP_014683038.1	100	NA	NA	WP_014333903.1	98
*nifB*	FeMo cofactor biosynthesis protein NifB	JHW33_RS22770	WP_200227556.1	NA	NA	WP_014683037.1	99	NA	NA	WP_014333904.1	98
**Maturation of Nitrogenase**											
*nifM*	Nitrogen fixation protein NifM	JHW33_RS22785	WP_014683034.1	NA	NA	WP_014683034.1	100	NA	NA	WP_014333907.1	97
*nifZ*	Nitrogen fixation protein NifZ	JHW33_RS22790	WP_014683033.1	NA	NA	WP_014683033.1	100	NA	NA	WP_014333908.1	97
**Electron Transport**											
*nifF*	Flavodoxin I	JHW33_RS22760	WP_014683039.1	NA	NA	WP_014683039.1	100	NA	NA	WP_014333902.1	96
*nifJ*	Pyruvate ferredoxin/flavodoxin oxidoreductase	JHW33_RS06430	WP_200225589.1	WP_119261595.1	99	WP_013575392.1	99	WP_013575392.1	99	WP_015697263.1	98
**Regulation of nif Gene Expression**											
*nifL*	Nitrogen fixation regulatory protein NifL	JHW33_RS22780	WP_200227570.1	NA	NA	WP_014683035.1	99	NA	NA	WP_014333906.1	98
*nifA*	nif-specific transcriptional activator NifA	JHW33_RS22775	WP_014683036.1	NA	NA	WP_014683036.1	100	NA	NA	WP_014333905.1	99
**Unknown Function**											
*nifT*	Nitrogen fixation protein NifT	JHW33_RS22840	WP_014333918.1	NA	NA	WP_014333918.1	100	NA	NA	WP_014333918.1	100
*nifW*	Nif-specific regulatory protein NifW	JHW33_RS22795	WP_014683032.1	NA	NA	WP_014683032.1	100	NA	NA	WP_014333909.1	97
*nifI*	Hypothetical protein	JHW33_RS22815	WP_014683028.1	NA	NA	WP_014683028.1	100	NA	NA	WP_014333913.1	97
**Nitrogen Regulation Related Genes**											
*glnQ*	Glutamine ABC transporter ATP-binding protein	JHW33_RS01275	WP_013574681.1	WP_013574681.1	100	WP_013574681.1	100	WP_013574681.1	100	WP_015696540.1	99
*ntrB*	Nitrogen regulation protein NR(II)	JHW33_RS04965	WP_156106968.1	WP_112152400.1	99	WP_013577653.1	99	WP_013575697.1	99	WP_015697258.1	96
*nac*	Nitrogen assimilation transcriptional regulator	JHW33_RS02570	WP_013574993.1	WP_119261788.1	100	WP_015689657.1	99	WP_013574993.1	99	WP_037038858.1	95
*glnB*	nitrogen regulatory protein P-II	JHW33_RS21825	WP_013574351.1	WP_013574351.1	100	WP_013573506.1	100	WP_013574351.1	100	WP_013574351.1	99
*glnD*	PII uridylyl-transferase	JHW33_RS20990	WP_013574191.1	WP_013574191.1	100	WP_013574191.1	100	WP_013574191.1	100	WP_015696089.1	99
*glnA*	Glutamine synthetase	JHW33_RS16330	WP_013577652.1	WP_013577652.1	100	WP_015690611.1	100	WP_013577652.1	99	WP_013577652.1	98
*glnG*	Nitrogen regulation protein NR(I)	JHW33_RS16340	WP_013577654.1	WP_013577654.1	100	WP_013577654.1	100	WP_013577654.1	100	WP_015699252.1	99

*ND, not determined; NA, not available.*

#### Production of the Plant Growth Hormone Indole-3-Acetic Acid

The comparative genome analysis indicated that eleven key genes responsible for IAA production were found in the ZF458 genome, which contained eight genes related to tryptophan operon (*trp*G, *trp*E, *trp*D, *trp*CF, *trp*B, *trp*A, *trp*S, and *trp*R), *mtr* gene encoding tryptophan permease, *ipd*C gene encoding indolepyruvate decarboxylase, and *acd*S gene encoding 1-aminocyclopropane-1-carboxylate (ACC) deaminase. Moreover, all of these genes were found in the genomes of ZF458, ZF7, HX2, Y9602 and ATCC 33071^T^ with sequence identities exceeding 93% at the amino acid level (Supplementary Table 3).

#### Phosphate Solubilization

According to the comparative genomic analysis, 26 genes involved in phosphate solubilization were found in the ZF458 genome. Among these genes, six genes (*aph*A, *pho*N, *iap*, *pho*A, *pho*B, and *pho*R) were related to organic phosphate acquisition, and twenty genes were associated with carbon-phosphorus lyase, which contained the whole carbon-phosphorus lyase operon (*phn*NMLKJIHGFAEDC) and phosphate transporter (*pst*SCAB) (Supplementary Table 4). All these genes retrieved from the ZF458 genome shared high similarities (greater than 94%) with those of four other *Rahnella* strains ZF7, HX2, Y9602 and ATCC 33071, except the gene *aph*A, which was absent in the ATCC 33071 genome (Supplementary Table 4).

#### Biosynthesis of Organic Acid

Furthermore, 44 genes linked with organic acid biosynthesis were found in the genome of strains ZF458, ZF7, HX2, and Y9602, while three genes *eda*, *idn*, and *ldh* were absent in the genome of ATCC 33071 (Table 4). The 44 genes were assigned for diverse pathways, including 3 genes for Glycolytic pathway (EMP), 24 genes for Tricarboxylic Acid Cycle pathway (TCA), 4 genes for Entner-Doudoroff pathway (ED), 3 genes for direct oxidation pathway, and 10 genes for another undefined pathway. The identities of these genes between ZF458 and ZF7, HX2, Y9602 were higher than those between ZF458 and ATCC 33071^T^ strains, especially for the gene *gnt*K, which only showed 52% sequence identities between ZF458 and ATCC 33071^T^ (Table 4). The results demonstrated that these strains may play important roles in inorganic phosphorus dissolution.

**TABLE 4:** Homolog analysis of organic acid biosynthesis genes in *R. aceris* ZF458 and other *Rahnella* strains.

Strain		*Rahnella aceris* ZF458		*R. aquatilis* ZF7		*R. aquatilis* HX2		*Rahnella* sp. Y9602		*R. aquatilis* ATCC 33071	
Genes	Product definition	Locus Tag	Protein ID	Protein ID	Homology (%)	Protein ID	Homology (%)	Protein ID	Homology (%)	Protein ID	Homology (%)
**EMP Pathway**											
*glk*	Glucokinase	JHW33_RS00015	WP_200224559.1	WP_013574458.1	100	WP_013574458.1	99	WP_013574458.1	99	WP_015696339.1	97
*zwf*	Glucose-6-phosphate dehydrogenase	JHW33_RS06770	WP_200225846.1	WP_013575315.1	100	WP_013575315.1	99	WP_013575315.1	99	WP_015697179.1	99
*ppc*	Phosphoenolpyruvate carboxylase	JHW33_RS15915	WP_013577573.1	WP_013577573.1	100	WP_013577573.1	100	WP_013577573.1	100	WP_015699176.1	99
**TCA Pathway**											
*gltA*	Citrate synthase	JHW33_RS09960	WP_013576444.1	WP_013576444.1	100	WP_013576444.1	100	WP_013576444.1	100	WP_015698117.1	99
*acnB*	Aconitate hydratase 2	JHW33_RS12660	WP_013576973.1	WP_013576973.1	100	WP_013576973.1	100	WP_013576973.1	100	WP_015698635.1	99
*acnA*	Aconitate hydratase	JHW33_RS04170	WP_037034468.1	WP_013575910.1	100	WP_015690067.1	99	WP_013575910.1	99	WP_015697115.1	98
*icd*	NADP-dependent Isocitrate dehydrogenase	JHW33_RS08805	WP_013576210.1	WP_013576210.1	100	WP_013576210.1	100	WP_013576210.1	100	WP_015697904.1	99
*aceK*	bifunctional isocitrate dehydrogenase	JHW33_RS14895	WP_200223135.1	WP_112197688.1	99	WP_015690563.1	99	WP_013577389.1	98	WP_015699020.1	95
*icl*	isocitrate lyase/phosphoenolpyruvate mutase family protein	JHW33_RS17355	WP_037033767.1	WP_119261086.1	99	WP_013573492.1	99	WP_013573492.1	99	WP_014333435.1	98
*sucA*	2-ketoglutarate dehydrogenase E1 component	JHW33_RS09935	WP_013576439.1	WP_119261848.1	100	WP_013576439.1	100	WP_013576439.1	100	WP_015698113.1	99
*odhB*	2-oxoglutarate dehydrogenase complex	JHW33_RS09930	WP_013576438.1	WP_013576438.1	100	WP_013576438.1	100	WP_013576438.1	100	WP_015698112.1	99
*sucC*	Succinyl-CoA synthetase beta subunit	JHW33_RS09925	WP_200227035.1	WP_013576437.1	99	WP_013576437.1	99	WP_013576437.1	99	WP_013576437.1	99
*sucD*	Succinyl-CoA synthetase alpha subunit	JHW33_RS09920	WP_013576436.1	WP_013576436.1	100	WP_013576436.1	100	WP_013576436.1	100	WP_013576436.1	100
*sdhA*	Succinate dehydrogenase flavoprotein subunit	JHW33_RS09945	WP_013576441.1	WP_013576441.1	100	WP_013576441.1	100	WP_013576441.1	100	WP_015698115.1	99
*sdhB*	succinate dehydrogenase iron-sulfur subunit	JHW33_RS09940	WP_013576440.1	WP_013576440.1	100	WP_013576440.1	100	WP_013576440.1	100	WP_015698114.1	99
*sdhC*	Succinate dehydrogenase cytochrome b556 large membrane subunit	JHW33_RS09955	WP_013576443.1	WP_013576443.1	100	WP_013576443.1	100	WP_013576443.1	100	WP_015698116.1	99
*sdhD*	succinate dehydrogenase membrane anchor subunit	JHW33_RS09950	WP_013576442.1	WP_013576442.1	100	WP_013576442.1	100	WP_013576442.1	100	WP_013576442.1	100
*fumA/B*	Fumarate hydratase, class I	JHW33_RS24430	WP_013578285.1	WP_013578285.1	100	WP_013578285.1	100	WP_013578285.1	100	WP_014341906.1	99
*fumC*	Fumarate hydratase, class II	JHW33_RS05805	WP_200225335.1	WP_112152260.1	99	WP_013575516.1	99	WP_013575516.1	99	WP_015697345.1	99
*frdA*	Fumarate reductase flavoprotein subunit	JHW33_RS18705	WP_014411553.1	WP_119261153.1	100	WP_014411553.1	100	WP_013573749.1	100	WP_014333648.1	99
*frdB*	succinate dehydrogenase/fumarate reductase iron-sulfur subunit	JHW33_RS18700	WP_013573748.1	WP_119261152.1	100	WP_013573748.1	100	WP_013573748.1	100	WP_014333647.1	97
*frdC*	Fumarate reductase subunit C	JHW33_RS18695	WP_200223783.1	WP_119261151.1	99	WP_013573747.1	99	WP_013573747.1	99	WP_014333646.1	97
*frdD*	Fumarate reductase subunit D	JHW33_RS18690	WP_014333645.1	WP_013573746.1	100	WP_013573746.1	100	WP_013573746.1	99	WP_014333645.1	99
*mdh*	Malate dehydrogenase	JHW33_RS18970	WP_013573800.1	WP_037034246.1	100	WP_013573800.1	100	WP_013573800.1	100	WP_014333686.1	97
*maeB*	NADP-dependent oxaloacetate-decarboxylating malate dehydrogenase	JHW33_RS22145	WP_013574414.1	WP_013574414.1	100	WP_013574414.1	100	WP_013574414.1	100	WP_015696301.1	99
*aceA*	Isocitrate lyase	JHW33_RS14900	WP_013577390.1	WP_013577390.1	100	WP_013577390.1	100	WP_013577390.1	100	WP_015699021.1	98
*aceB/glcB*	Malate synthase	JHW33_RS14905	WP_200223138.1	WP_119262163.1	99	WP_013577391.1	99	WP_013577391.1	99	WP_015699022.1	96
**ED Pathway**											
*gntk*	gluconokinase	JHW33_RS05130	WP_200225227.1	WP_015689980.1	99	WP_015689980.1	99	WP_015689980.1	98	WP_013573544.1	52
*edd*	phosphogluconate dehydratase	JHW33_RS05140	WP_200225228.1	WP_119261688.1	99	WP_015689978.1	99	WP_013575660.1	99	WP_015697147.1	95
*eda*	bifunctional 4-hydroxy-2-oxoglutarate aldolase/2-dehydro-3-deoxy-phosphogluconate aldolase	JHW33_RS05145	WP_037035690.1	WP_013575316.1	99	WP_013575316.1	99	WP_013575316.1	99	WP_015697180.1	91
*eda*	bifunctional 4-hydroxy-2-oxoglutarate aldolase/2-dehydro-3-deoxy-phosphogluconate aldolase	JHW33_RS06765	WP_013575316.1	WP_112198389.1	100	WP_013575659.1	100	WP_013575659.1	100	NA	NA
**Direct Oxidation Pathway**											
*gcd*	glucose/quinate/shikimate family membrane-bound PQQ-dependent dehydrogenase	JHW33_RS25310	WP_013578038.1	WP_112197657.1	100	WP_013578038.1	100	WP_013578038.1	100	WP_014341759.1	97
*idn*	gluconate 5-dehydrogenase	JHW33_RS05495	WP_200225254.1	WP_119261658.1	87	WP_013575578.1	87	WP_013575578.1	87	NA	NA
*idnO*	Gluconate-5-dehydrogenase	JHW33_RS01075	WP_037036159.1	WP_112151190.1	99	WP_013574641.1	99	WP_013574641.1	99	WP_015696505.1	99
**Other Pathway**											
*ldh*	L-lactate dehydrogenase	JHW33_RS10455	WP_200227073.1	WP_112151693.1	99	WP_013576533.1	99	WP_013576533.1	99	NA	NA
*aceE*	pyruvate dehydrogenase (acetyl-transferring), homodimeric type	JHW33_RS12730	WP_013576985.1	WP_013576985.1	100	WP_013576985.1	100	WP_013576985.1	100	WP_015698648.1	99
*aceF*	Pyruvate dehydrogenase E2 component	JHW33_RS12725	WP_200222913.1	WP_015690459.1	99	WP_015690459.1	99	WP_013576984.1	99	WP_015698647.1	99
*pta*	Phosphate acetyltransferase	JHW33_RS00765	WP_153374992.1	WP_153374992.1	100	WP_153374992.1	100	WP_153374992.1	100	WP_193785502.1	99
*ackA*	Acetate kinase A	JHW33_RS00770	WP_013574580.1	WP_013574580.1	100	WP_015689524.1	100	WP_013574580.1	99	WP_015696452.1	99
*poxB*	Pyruvate dehydrogenase	JHW33_RS01625	WP_200224987.1	WP_112198008.1	99	WP_013574762.1	99	WP_013574762.1	99	WP_015696595.1	99
*pflA*	Pyruvate formate lyase activating enzyme I	JHW33_RS01755	WP_015689580.1	WP_015689580.1	100	WP_015689580.1	100	WP_015689580.1	100	WP_015689580.1	100
*pflC*	formate-C-acetyltransferase-activating enzyme	JHW33_RS15250	WP_200223220.1	WP_037035395.1	99	WP_015690573.1	99	WP_013577447.1	99	WP_015699070.1	89
*pflB*	formate C-acetyltransferase	JHW33_RS01760	WP_013574828.1	WP_013574828.1	100	WP_013574828.1	100	WP_013574828.1	100	WP_013574828.1	100
*pflE*	glycyl-radical enzyme activating protein	JHW33_RS04230	WP_200225174.1	WP_015690059.1	99	WP_015690059.1	99	WP_013575898.1	99	WP_015697125.1	94

*ND, not determined; NA, not available.*

#### Production of Plant Promotion Hormone Pyrroloquinoline Quinone

According to the comparative analysis, the *R. aceris* ZF458 plasmid genome contained a highly conserved pyrroloquinoline quinone gene cluster (*pqq*ABCDEF) and upstream promoter *orf*X, covering 6,631 bp with 7 ORFs (Supplementary Figure 7), and the *gcd* gene encoding PQQ-dependent dehydrogenase. The *pqq* gene cluster of ZF458 shared a high homology with those in other four *Rahnella* strains (>95% identity), except that the sequence identity of *pqq*F between ZF458 and ATCC 33071^T^ was only 80% (Supplementary Table 5).

### Survival and Adapting to Environmental Variations

#### Bacterial Secretion System

##### Type II Secretion System

**FIGURE 4:**
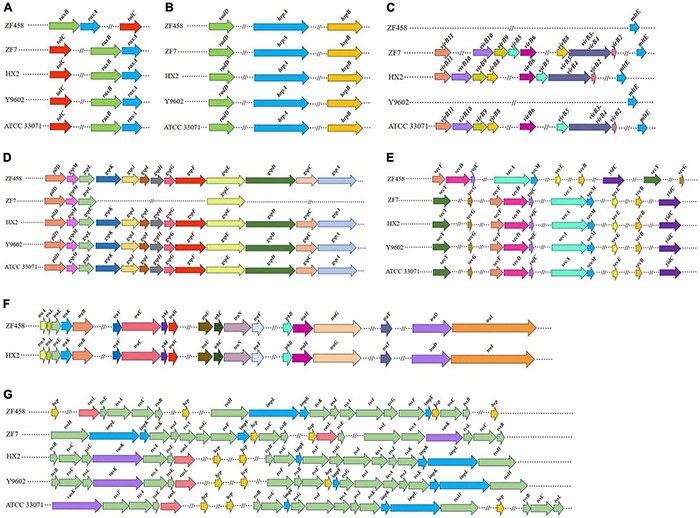
Comparison of the bacterial secretion system gene clusters of *R. aceris* ZF458 against four previously fully sequenced *Rahnella* genomes. **(A)** Type I secretion system. **(B)** Type III secretion system. **(C)** Type IV secretion system. **(D)** Type II secretion system. **(E)** Sec (secretion) system. **(F)** Conjugal transfer region of type IV secretion. **(G)** Type VI secretion system. The same color represented genes with the same or similar biological function. Arrows denoted putative transcriptional units. The length of blocks represented the size of genes (1 cm = 1000 bp).

In order to explore the different secretion systems, five released complete genome sequences of *Rahnella* strains namely ZF458, ZF7, HX2, Y9602, ATCC 33071^T^ were compared. The genome of ZF458 contained a variety of secretion systems, which were closely associated with the life activities of bacteria. The comparative analysis showed that the *R. aceris* ZF458 chromosome contained a highly conserved T2SS (type II secretion system) gene cluster (*gsp*ACDEFGHIJKLM and *pil*D) (Figure 4D), covering 12,797 bp with 13 ORFs. All 13 genes existed in strains ZF458, HX2, Y9602 and ATCC 33071^T^, while only four genes (*gsp*E, *gsp*L, *gsp*M, and *pil*D) were found in ZF7 genome. The *gsp* gene cluster from the ZF458 genome shared a high consistency with strains HX2 and Y9602 (exceeding 97%) at the amino acid level, whereas lower sequence identities for genes *gsp*A, *gsp*C, *gsp*I, *gsp*J, *gsp*L, and *gsp*M (under 80%) were found between ZF458 and ATCC 33071^T^ (Supplementary Table 6).

##### Type IV Secretion System

In addition, nine key genes related to T4SS (*vir*B2,3-4,5,6,8,9,10,11) were found in strains ZF7, HX2, and ATCC 33071^T^, but not in ZF458 and Y9602 (Figure 4C), and all the genes were retrieved from the chromosome genome. However, another gene *mlt*E associated with T4SS was found in the five *Rahnella* strains and shared a 99% similarity at the amino acid level (Supplementary Table 6). Furthermore, the *tra* and *trb* gene clusters with 19 ORFs associated with the conjugal transfer region of the type IV secretion system were discovered in the plasmid genomes of ZF458 and HX2 (Figure 4F). Most of these genes were highly conserved with sequence identities exceeding 90%, except that *tra*G and *tra*I with sequence identities 87 and 89%, respectively (Supplementary Table 6). Interestingly, none of these genes existed in the genome of strains ZF7, Y9602 and ATCC 33071^T^.

##### Type VI Secretion System

In this study, 27 genes related to T6SS were found in the genome of *R. aceris* ZF458, and 13 genes were regarded as core genes (Figure 4G). The 13 core T6SS genes were highly conserved in the five *Rahnella* strains with a consistency of amino acid sequences over 90%, although the genes were in different encoding orders. However, 14 core T6SS genes existed in the genome of strains ZF7, HX2, Y9602, and ATCC 33071^T^, for the core gene *tssL* absent in the ZF458 genome (Supplementary Table 6). Moreover, other function genes involved in inner membrane proteins (ImpL and ImpK), extracellular structural components *(vgr*G and *hcp*) and regulatory proteins or structural proteins (VasL and ImpF) were present in the ZF458 genome, and shared a high similarity with those of the other 4 *Rahnella* strains at the amino acid level (Supplementary Table 6).

##### Other Secretion Systems

In addition, three genes related to T1SS (*rax*A, *rax*B, and *tol*C) (Figure 4A), three genes associated with T3SS (*hrp*A, *hrp*B, and *rad*D) (Figure 4B), ten genes associated with Sec system (Figure 4E), four genes connected with twin arginine targeting (*tat*B, *tat*C, and *tat*E) and two genes related to SRP components (*ffh* and *fts*Y) were all present in the genomes of ZF458, ZF7, HX2, Y9602 and ATCC 33071^T^, most of which were highly conserved (Supplementary Table 6).

#### Two-Component System

Moreover, 19 TCSs (two-component system) were found in the genomes of strains ZF458, ZF7, HX2, Y9602 and ATCC 33071^T^, most of which were highly conserved (Supplementary Table 7). According to the homology, the topological characteristics of sensor histidine kinase (HK) and response regulator (RR) (Lavín et al., 2007), the 19 TCSs were divided into diverse subfamilies. For insurance, the motility TCS (*Che*A/Y) existed in ZF458 with a high identity (more than 98%) at the amino acid level among of the five *Rahnella* strains. The nitrogen regulation TCS (*gln*L/G) was searched for in ZF458 and shared a high homology with that in ZF7, HX2, Y9602 and ATCC 33071^T^ (>99% identity). Phosphate regulation TCS (*pho*R/B) was observed in the genomes of strains ZF458, ZF7, HX2, Y9602 and ATCC 33071^T^, with sequence identities exceeding 98% (Supplementary Table 7). All these genes might play important roles in *R. aceris* ZF458 in terms of physiological metabolism and environmental adaptation.

#### Selenium Metabolism

In addition, many genes associated with selenium metabolism were found in the genome of *R. aceris* ZF458, which were similar to strains ZF7, HX2, Y9602 and ATCC 33071^T^ (identities >90%) (Table 5). Among these genes, 8 genes including *cys*A, *cys*W, *cys*T, *cys*P, *cys*J, *cys*I, *tsg*A, and *hfq* were associated with selenium transportation and regulation, 19 genes containing *nar* gene cluster and *tat* gene cluster were associated with selenate reduction, 15 genes were related to selenite reduction, and 8 genes were involved in selenium association (Table 5).

**TABLE 5:** Genes related to selenium metabolism in *R. aceris* ZF458 and other *Rahnella* strains.

Strain		*Rahnella aceris* ZF458		*R. aquatilis* ZF7		*R. aquatilis* HX2		*Rahnella* sp. Y9602		*R. aquatilis* ATCC 33071	
Genes	Product definition	Locus Tag	Protein ID	Protein ID	Homology (%)	Protein ID	Homology (%)	Protein ID	Homology (%)	Protein ID	Homology (%)
**Selenium transportation**											
*cysA*	sulfate/thiosulfate ABC transporter ATP-binding protein CysA	JHW33_RS22210	WP_013574427.1	WP_013574427.1	100	WP_013574427.1	100	WP_013574427.1	100	WP_015696312.1	98
*cysW*	sulfate/thiosulfate ABC transporter permease CysW	JHW33_RS22205	WP_013574426.1	WP_013574426.1	100	WP_013574426.1	100	WP_013574426.1	100	WP_015696311.1	97
*cysT*	sulfate/thiosulfate ABC transporter permease CysT	JHW33_RS22200	WP_013574425.1	WP_013574425.1	100	WP_013574425.1	100	WP_013574425.1	100	WP_013574425.1	100
*cysP*	thiosulfate-binding protein	JHW33_RS22195	WP_200224495.1	WP_112151264.1	99	WP_013574424.1	99	WP_013574424.1	99	WP_015696310.1	97
*cysJ*	NADPH-dependent assimilatory sulfite reductase flavoprotein subunit	JHW33_RS19955	WP_037034152.1	WP_119261245.1	99	WP_013574003.1	99	WP_013574003.1	99	WP_015695893.1	98
*cysI*	assimilatory sulfite reductase (NADPH) hemoprotein subunit	JHW33_RS19960	WP_200227283.1	WP_119262218.1	99	WP_013574004.1	99	WP_013574004.1	99	WP_015695894.1	98
*tsgA*	MFS transporter TsgA	JHW33_RS01475	WP_200224981.1	WP_013574731.1	99	WP_015689560.1	99	WP_013574731.1	99	WP_015696567.1	97
*hfq*	RNA chaperone Hfq	JHW33_RS18790	WP_013573763.1	WP_013573763.1	100	WP_013573763.1	100	WP_013573763.1	100	WP_013573763.1	100
**Selenate reduction**											
*narG*	nitrate reductase subunit alpha	JHW33_RS04765	WP_200225200.1	WP_112152550.1	99	WP_013575749.1	99	WP_013575749.1	99	WP_015697516.1	99
*narH*	nitrate reductase subunit beta	JHW33_RS04770	WP_200225201.1	WP_037034411.1	99	WP_013575748.1	99	WP_013575748.1	99	WP_015697515.1	99
*narJ*	nitrate reductase molybdenum cofactor assembly chaperone	JHW33_RS04775	WP_013575747.1	WP_013575747.1	100	WP_013575747.1	100	WP_013575747.1	100	WP_015697514.1	94
*narI*	respiratory nitrate reductase subunit gamma	JHW33_RS04780	WP_013575746.1	WP_013575746.1	100	WP_013575746.1	100	WP_013575746.1	100	WP_015697514.1	99
*narX*	nitrate/nitrite two-component system sensor histidine kinase NarX	JHW33_RS06285	WP_131637636.1	WP_013575417.1	99	WP_015689909.1	99	WP_013575417.1	99	WP_015697474.1	96
*narL*	two-component system response regulator NarL	JHW33_RS06290	WP_013575416.1	WP_013575416.1	100	WP_013575416.1	100	WP_013575416.1	100	WP_015697475.1	99
*fdnG*	formate dehydrogenase-N subunit alpha	JHW33_RS16845	WP_200223551.1	WP_119261065.1	99	WP_013573395.1	99	WP_013573395.1	99	WP_014333323.1	99
*dmsB*	dimethylsulfoxide reductase subunit B	JHW33_RS01730	WP_013574782.1	WP_013574782.1	100	WP_013574782.1	100	WP_013574782.1	100	WP_015696614.1	99
*dmsA*	dimethylsulfoxide reductase subunit A	JHW33_RS01725	WP_037036060.1	WP_112198016.1	99	WP_013574781.1	99	WP_013574781.1	99	WP_015696613.1	98
*dmsD*	Tat proofreading chaperone DmsD	JHW33_RS01740	WP_037036059.1	WP_112151145.1	99	WP_013574784.1	99	WP_013574784.1	99	WP_015696616.1	95
*dmsC*	dimethyl sulfoxide reductase anchor subunit family protein	JHW33_RS01735	WP_013574783.1	WP_013574783.1	100	WP_013574783.1	100	WP_013574783.1	100	WP_015696615.1	94
*ynfE*	dimethyl sulfoxide reductase subunit A	NA	NA	WP_119261427.1	NA	WP_013574780.1	NA	WP_013574780.1	NA	WP_015696612.1	NA
*arrA*	molybdopterin-dependent oxidoreductase	JHW33_RS25040	WP_013578099.1	WP_119262333.1	100	WP_013578099.1	100	WP_013578099.1	100	WP_014341807.1	94
*tatE*	twin-arginine translocase subunit TatE	JHW33_RS10290	WP_013576504.1	WP_013576504.1	100	WP_013576504.1	100	WP_013576504.1	100	WP_013576504.1	100
*tatE*	twin-arginine translocase subunit TatE	JHW33_RS15180	WP_013577433.1	WP_013577433.1	100	WP_013577433.1	100	WP_013577433.1	100	WP_013577433.1	100
*tatB*	Sec-independent protein translocase subunit TatB	JHW33_RS15175	WP_013577432.1	WP_013577432.1	100	WP_013577432.1	100	WP_013577432.1	100	WP_015699057.1	90
*tatC*	Sec-independent protein translocase subunit TatC	JHW33_RS15170	WP_013577431.1	WP_013577431.1	100	WP_013577431.1	100	WP_013577431.1	100	WP_013577431.1	100
*tatD*	3′-5′ ssDNA/RNA exonuclease TatD	JHW33_RS15165	WP_037035383.1	WP_119262167.1	100	WP_015690569.1	100	WP_013577430.1	100	WP_015699056.1	100
*fnr*	FNR family transcription factor	JHW33_RS05565	WP_013575564.1	WP_013575564.1	100	WP_013575564.1	100	WP_013575564.1	100	WP_013575564.1	100
**Selenite reduction**											
*nirB*	nitrite reductase large subunit NirB	JHW33_RS04975	WP_200225216.1	WP_119262240.1	99	WP_013575696.1	99	WP_013575695.1	99	WP_015697260.1	96
*nirD*	nitrite reductase large subunit NirD	JHW33_RS17985	WP_200223720.1	WP_119261122.1	97	WP_013573623.1	97	WP_013573623.1	97	WP_037040691.1	96
*frdA*	flavocytochrome c	JHW33_RS05300	WP_134705689.1	WP_013575619.1	99	WP_013575619.1	99	WP_013575619.1	99	WP_015697429.1	99
*frdD*	fumarate reductase subunit FrdD	JHW33_RS18690	WP_014333645.1	WP_013573746.1	100	WP_013573746.1	100	WP_013573746.1	99	WP_014333645.1	99
*frdC*	fumarate reductase subunit FrdC	JHW33_RS18695	WP_200223783.1	WP_119261151.1	99	WP_013573747.1	99	WP_013573747.1	99	WP_014333646.1	97
*frdA*	fumarate reductase (quinol) flavoprotein subunit	JHW33_RS18705	WP_014411553.1	WP_119261153.1	100	WP_014411553.1	99	WP_013573749.1	99	WP_014333648.1	99
*iscR*	Fe-S cluster assembly transcriptional regulator IscR	JHW33_RS21885	WP_013574363.1	WP_013574363.1	100	WP_013574363.1	100	WP_013574363.1	100	WP_015696248.1	99
*gorA*	glutathione-disulfide reductase	JHW33_RS17480	WP_037033742.1	WP_119261551.1	99	WP_013573520.1	99	WP_013573520.1	99	WP_014333456.1	99
*trxA*	thioredoxin TrxA	JHW33_RS15705	WP_013577537.1	WP_013577537.1	100	WP_013577537.1	100	WP_013577537.1	100	WP_013577537.1	100
*trxB*	thioredoxin-disulfide reductase	JHW33_RS01690	WP_015689578.1	WP_015689578.1	100	WP_015689578.1	100	WP_013574774.1	99	WP_015696607.1	99
*trxC*	thioredoxin TrxC	JHW33_RS20360	WP_013574074.1	WP_013574074.1	100	WP_013574074.1	100	WP_013574074.1	100	WP_015695963.1	99
*grxA*	GrxA family glutaredoxin	JHW33_RS04425	WP_013575818.1	WP_013575818.1	100	WP_013575818.1	100	WP_013575818.1	100	WP_013575818.1	100
*grxB*	glutaredoxin 2	JHW33_RS02700	WP_013575015.1	WP_013575015.1	100	WP_013575015.1	100	WP_013575015.1	100	WP_015696848.1	95
*grxC*	glutaredoxin 3	JHW33_RS16070	WP_013577603.1	WP_013577603.1	100	WP_013577603.1	100	WP_013577603.1	100	WP_015699202.1	99
*sodA*	superoxide dismutase, Mn	JHW33_RS16835	WP_013573393.1	WP_013573393.1	100	WP_013573393.1	100	WP_013573393.1	100	WP_014333320.1	98
**Selenium association**											
*selA*	DgaE family pyridoxal phosphate-dependent ammonia lyase	JHW33_RS14260	WP_200223032.1	WP_013577265.1	99	WP_013577265.1	99	WP_013577265.1	99	WP_015698901.1	97
*cysK*	cysteine synthase A	JHW33_RS22245	WP_013574434.1	WP_013574434.1	100	WP_013574434.1	100	WP_013574434.1	100	WP_015696317.1	99
*metB*	cystathionine gamma-synthase	JHW33_RS15930	WP_013577576.1	WP_013577576.1	100	WP_013577576.1	100	WP_013577576.1	100	WP_015699179.1	99
*metC*	cystathionine beta-lyase	JHW33_RS19835	WP_200224006.1	WP_119261241.1	99	WP_013573978.1	99	WP_013573978.1	99	WP_015695874.1	96
*metE*	5-methyltetrahydropteroyl- triglutamate-homocysteine S-methyltransferase	JHW33_RS15280	WP_200223241.1	WP_119262170.1	99	WP_013577450.1	99	WP_013577450.1	99	WP_015699072.1	97
*metH*	methionine synthase	JHW33_RS14885	WP_200223128.1	WP_013577387.1	99	WP_013577387.1	99	WP_013577387.1	99	WP_015699018.1	99
*sufS*	cysteine desulfurase SufS	JHW33_RS07945	WP_200226532.1	WP_112197126.1	99	WP_013576097.1	99	WP_013576097.1	99	WP_015697804.1	97
*sufE*	cysteine desulfuration protein SufE	JHW33_RS07940	WP_134705911.1	WP_112152177.1	98	WP_013576096.1	98	WP_013576096.1	98	WP_015697803.1	96

*ND, not determined; NA, not available.*

#### Acid Resistance

*Rahnella* strains adapted to diverse ecological environments, including water, soil and plants, which may be attributed to their stress tolerance traits. In this study, 28 genes associated with acid resistance were present in ZF458, and were grouped into different systems according to the enzyme activity. All the genes shared high similarities (greater than 90%) with those of other *Rahnella* strains, except for the gene *omp*F (encoding porin), which showed a lower homology with ZF7, HX2, Y9602 and ATCC 33071^T^ (identity <90%) (Supplementary Table 8).

#### Motility, Chemotaxis and Quorum Sensing

A group of tightly gene clusters (*flh*, *flg*, and *fli*) related to flagella biosynthesis encoding 41 flagella-associated proteins (FlhDC, MotAB, FlhBAE, FlgA-N, FliC-T, FliA, and FliZ) were found in the genome of *R. aceris* ZF458, and had high amino acid similarities among the five *Rahnella* strains, except for the gene *fli*C, and *fli*D, which displayed low similarities between ZF458 and ATCC 33071^T^ (49% identities) (Supplementary Table 9). In addition, 8 genes (*che*A, *che*W, *mcp*, *tar*, *che*R, *che*B, *che*Y, and *che*Z) associated with chemotaxis were found in the genome of ZF458, with sequence similarities exceeding 97% between ZF458 and other four *Rahnella* strains (Supplementary Table 9). 12 key genes related to the autoinducer-2 (AI-2)- dependent signaling systems were detected in the five *Rahnella* strains, with sequences identities exceeding 94% between ZF458 and four other strains ZF7, HX2, Y9602 and ATCC 33071 (Supplementary Table 10).

## Discussion

*Rahnella* was widely distributed in a variety of environments, and had been reported as PGPR that benefited plant growth (Sallam, 2011; Li et al., 2014). However, their specific mechanisms of plant-growth promotion and environmental adaption at the molecular level especially for *R. aceris* were still unclear. In this study, *R. aceris* ZF458 which was isolated from swamp soil was displayed. Strain ZF458 produced siderophores and phosphatases, which were key factors to promote plant growth (Li et al., 2013). ZF458 presented broad spectrum antagonistic activities against various plant pathogens and exhibited a significant effect in controlling *Agrobacterium tumefaciens* on sunflowers. All of these characteristics indicated that ZF458 might be a plant growth-promoting rhizobacteria. The complete genome of *R. aceris* ZF458 was sequenced and compared with other *Rahnella* strains to better understand the molecular mechanisms for plant-growth promotion and environmental adaption.

The phylogenetic trees showed that ZF458 clustered together with SAP-19, and the two strains both belonged to the *R. aceris* species, forming a tight cluster with *R. aquatilis*, which was consistent with a previous study (Lee et al., 2020). A previous study also indicated that *R. aceris* SAP-19 shared an ANI value of 92.7% and a DDH value under 48.6% with the genome sequence of *R. aquatilis* (Lee et al., 2020), however, our study showed that higher ANI and DDH values (>96and >70%, respectively) were calculated between *R. aceris* ZF458 and *R. aquatilis* strains. The results also indicated that strain ZF458 was closely related to the *R. aquatilis* strains, although it was classified as a new species of *Rahnella* genus by the phylogenetic analysis. According to the phylogenetic tree, strain ATCC 33071^T^ was in a farther clade. Interestingly, the analysis of ANI and DDH revealed the same result, as the ANI and DDH values between ZF458 and ATCC 33071^T^ were 92.75 and 49.4%. The results also indicated that the ANI and DDH values between ATCC 33071^T^ and other *R. aquatilis* strains (including HX2, ZF7, Y9602) were below 95 and 70%, respectively, and this was consistent with the previous report showing that the ANI value between *R. aquatilis* strains ZF7 and ATCC 33071^T^ was only 92.9% (Yuan et al., 2020). Previous studies indicated that strain ZF7, HX2 and Y9602 were both isolated from soil (Guo et al., 2012; Martinez et al., 2012a), while ATCC 33071^T^ was isolated from drinking water (Martinez et al., 2012b), so, it may suggest that the genetic evolutionary distance could be associated with the habitat and adaptation. All five *Rahnella* strains harbored one or more plasmids, which could be related to genetic evolution, based on the previous report that 19% of strains in the genus *Rahnella* were plasmid-containing and had highly homologous regions for the same species (Rozhon et al., 2010).

In the study, an obvious gene transfer between ZF458 and other *R. aquatilis* strains indicated that strain ZF458 was another species not belonging to *R. aquatilis*. Furthermore, the nucleotide level similarities between ZF458 and ZF7, HX2, Y9602 were markedly higher than the similarity between ZF458 and ATCC 33071^T^, demonstrating the far phylogenetic distance between ZF458 and ATCC 33071^T^ and supporting the phylogenetic result that ZF458 and ATCC 33071^T^ were in different subclades. Low similarity regions were found between ZF458 and SAP-19, although the two strains both belonged to the *R. aceris* species, for the reason of strain SAP-19 was just whole genome shotgun sequence, not completed genome sequence. Based on the comparative analysis, only one unique gene existed in the ZF458 genome, while no unique genes existed in ZF7, HX2, Y9602 and ATCC 33071^T^, which confirmed the phylogenetic analysis described above that ZF458 belonged to *R. aceris* but was closely clustered with *R. aquatilis*.

Nitrogen was an important element for plant growth since plants only absorbed reduced forms of nitrogen, such as ammonia and nitrates. Biological nitrogen fixation (BNF), a microbiological process which converted atmospheric nitrogen into a plant usable form, offered an alternative to N fertilizers (Brewin and Legocki, 1992). This capability presented an opportunity to improve the nitrogen source utilization and crop yields, through the introduction of nitrogen fixing bacteria into crops, or the nitrogenase enzyme responsible for nitrogen fixation (Oldroyd and Dixon, 2014). Recently, a limited number of archaea and bacteria were found to fix nitrogen, such as *Proteobacteria*, *Firmicutes*, *Cyanobacteria*, and *Actinobacteria* (Santos et al., 2012). *Rahnella* spp. were also reported to possess the capacity for nitrogen fixation (Berge et al., 1991). For, insurance, *R. aquatilis* HX2 was reported as a PGPR and could promote the growth of corn for the ability to fix nitrogen (Guo et al., 2012). Previous studies indicated that the *nif*HDK gene cluster encoding Mo-nitrogenase played important role in fixing nitrogen, and *nif*BENXV was important for the synthesis and maturation of the FeMo cofactor (Wang L. et al., 2013). In this study, 21 *nif* genes were detected in the genome of ZF458, which predicted that ZF458 had a strong nitrogen fixation ability.

Auxin IAA was a primary plant hormone synthesized by plant growth-promoting bacteria that had a profound influence on benefiting plant (Bal et al., 2013). Currently, *Rahnella* spp. isolated from different environments were reported to have a strong capacity to produce IAA. Such as *R. aquatilis* ZF7 exhibited a high IAA biosynthesis capacity of 30.86 μg mL^–1^ and had a significant growth promoting effect on Chinese cabbage (Yuan et al., 2020). *R. aquatilis* HX2 could biosynthesize IAA and significantly promote maize growth (Guo et al., 2012). The endophyte *Rahnella* strains isolated from sweet potato plants, showed the ability to produce IAA and revealed obvious growth promotion in hybrid poplar (Khan and Doty, 2009). In this study, 11 genes associated with IAA biosynthesis were found in the ZF458 genome, including 9 genes (*trp* gene cluster) linked with tryptophan, *ipd*C gene encoding indole-3-pyruvate decarboxylase, and *acd*S gene encoding ACC deaminase, which revealed high similarities to *R. aquatilis* strains HX2 and ZF7. Previous study showed that *acd*S gene played a major role in PGPR activities. For example, the disruption of *acdS* gene in *R. aquatilis* HX2 reduced IAA production and decreased the growth promotion activity on corn (Peng et al., 2019). Previous studies demonstrated that the IPyA pathway was the main IAA synthetic pathway in a broad range of bacteria (Eastman et al., 2014), and the *ipd*C gene played an important role in regulating the IPyA pathway (Spaepen et al., 2007). So, the IPyA pathway may play an important role in the biosynthesis of IAA for *R. aceris* ZF458.

Phosphorus was considered as an important macronutrient for the growth and development of plants. Microorganisms were reported to play an important role in dissolving the insoluble phosphates in the rhizosphere (Passariello et al., 2006). Currently, phosphate solubilization of microorganisms was usually divided into two aspects, inorganic phosphorus solubilization and organic phosphorus solubilization, and organic phosphate in soil was degraded mainly through enzymes such as phosphatase, phytase and carbon-phosphorus lyase (White and Metcalf, 2007). Previous study showed that the carbon-phosphorus lyase system which encoded by *phn* gene cluster played important roles in organic phosphate dissolution (Jochimsen et al., 2011). In this study, the entire *phn* gene cluster was observed in the ZF458 genome, which was highly conserved in *Rahnella* strains, thus indicating that strain ZF458 may possess the strong ability to dissolve organic phosphorus. In addition, mineral phosphate solubilization could be improved by the production of organic acids, including gluconic, formic, citric, acetic, lactic, and acetic (Vassilev et al., 2006). According to the comparative genomic analysis, 44 genes related to organic acid biosynthesis were retrieved in the ZF458 genome, which were highly similar to other *Rahnella* strains. Thus, the organic acids generated by these genes played an important role in inorganic phosphorus solubilization for *R. aceris* ZF458. Many *Rahnella* strains have been reported to have a strong phosphate-solubilizing activity or organic acid synthesis ability. For example, *R. aquatilis* JZ-GX1 was able to secrete organic acids in an iron-deficient environment, which facilitated the production of phosphatases and siderophores, and thus promoted plant growth (Kong et al., 2020b). *Rahnella* sp. W25 isolated from the crop rhizosphere of calcareous soil, showed the maximum phosphate-solubilizing capability on tricalcium phosphate, aluminum phosphate, and ferric phosphate of 385.5, 110.4, and 216.6 mg⋅L^–1^ respectively (Qiao et al., 2013). In addition, the phosphorous solubilized by *Rahnella* strains W25 was significantly negatively correlated with the pH of the culture medium (Qiao et al., 2013). The *aph*A gene encoded a molecular class B bacterial phosphatase was reported to exhibit a better activity at acidic pH values (Passariello et al., 2006). Importantly, the pH value of the liquid culture of *R. aceris* ZF458 reduced to 3.24 from the original value of 7.0 with the growth of culture time, which might indicate that the AphA (protein id, WP_134705824.1) enzyme generated strong activity when solubilizing organic phosphate.

Gluconic acid was reported as a crucial antibacterial substance produced by *R. aquatilis*, and pyrroloquinoline quinone (PQQ) was necessary for the production of gluconic acid (Choi et al., 2008). Previous studies had demonstrated that the GDH (glucose dehydrogenase)-PQQ holoenzyme was important for the biosynthesis of an antimicrobial substance (Matsushita et al., 2002; Werra et al., 2009), and PQQ was reported as a plant growth promotion factor, which in addition to gluconic acid production, has also been associated with its antioxidant properties (Choi et al., 2008). Bacterial genes associated with PQQ biosynthesis have been identified in various species were clustered in *pqq*ABCDEF operons (Guo et al., 2009). Previous studies revealed that *pqq*A gene encoded a small peptide that contained tyrosine and glutamate and served as the precursor and rate-determining step for PQQ production (Goosen et al., 1992). Also, *pqq*B was reported as a carrier that facilitated the secretion of PQQ across the plasma-membrane into the periplasm in *K. pneumoniae* (Velterop et al., 1995). For example, the disruption of the *pqq*A or *pqq*B gene in *R. aquatilis* HX2 reduced the plant growth promotion activity, and eliminated the ability to produce antibacterial substances thus decreasing the control effect against grapevine crown gall (Li et al., 2014). In this study, the whole *pqq* operons were observed in the ZF458 genome, and shared a high similarity with those in HX2, which indicated that *R. aceris* ZF458 could produce PQQ normally, although accurate biochemical functions remain to be confirmed.

PGPR exerted a series of regulatory mechanisms to adapt to the various complicated environments, among them, the bacterial secretion system, two-component system were well studied regulatory mechanisms associated with environmental adaption. Previous studies had indicated that large numbers of bacterial genomes (up to 17% in *Proteobacteria* genomes) encoded proteins in the general secretory pathway (GSP) (Bendtsen et al., 2005). Bacteria exported different kinds of substrates, including small molecules, extracellular enzymes, effector proteins and DNA *via* several sophisticated secretion systems, which were important for bacteria to adapt and survive the diverse ecological environments (Gerlach and Hensel, 2007).

Type II secretion system depended on the sec machinery and secreted folded proteins including various hydrolyzing enzymes such as the pseudolysin from the periplasm across the extracellular environment, which was important for bacterial survival in host or environment (Bendtsen et al., 2005). Many T2SS components had been well characterized, such as pectinases and cellulases associated with pathogenicity were secreted by T2SS in *Pectobacterium* (Filloux, 2004). T2SS was well-conserved and composed of 12–15 components, which were called Gsp proteins (Cianciotto, 2005). GspD formed a multimeric pore for transportation of secreted proteins and interacted with GspC, which played an important role in *V. cholerae* (Korotkov et al., 2011). The GspC, GspF, GspL, and GspM constituted the inner membrane platform and were associated with ATPase secretion in the cytoplasm (Johnson et al., 2006). In this study, ZF458 harbored 12 *gsp* genes which were highly conserved in the *Rahnella* strains, and the Gsps might be associated with the protein secretion and transportation, but the role of T2SS in *R. aceris* ZF458 needs to be determined in the future.

T4SS was characterized by the ability to regulate the translocation of single-stranded DNA or complex proteins, and existed in both Gram-negative and Gram-positive bacteria (Alvarez-Martinez and Christie, 2009). The T-DNA transfer system of *A. tumefaciens* was the prototypical type A T4SS, and together with the T4SS in *E. coli* was the best-characterized T4SS, which consisted of 12 proteins including VirB1-VirB11 and VirD4 (Christie et al., 2014). T4SS was recognized as the most ubiquitous secretion system in nature, for the ability to conjugate which was a common bacteria trait. However, no *vir* genes was found in the ZF458 genome, except for the conjugal transfer region (including *tra* and *trb* gene clusters) which existed in the plasmid genome. Previous studies revealed that T4SS evolved from bacterial conjugation machinery according to the sequence similarities (Christie and Cascales, 2003). Such as the octopine-type Ti-plasmids which were recognized as the conjugal transfer system in *Agrobacterium tumefaciens* was closely related to Ti plasmid *vir* genes (Alt-Mörbe et al., 1996). In addition, the *tra* genes related to conjugal transfer system were reported to be involved in the synthesis and possible retraction of the sex pilus (Winans and Walker, 1985), while the *vir* genes were also proven to be required for pilus biogenesis (Trokter et al., 2014). Thus, the gene clusters in the conjugal transfer region of ZF458 might possess the same function as T4SS, which needs to be further verified.

T6SS was reported to transfer effector proteins into eukaryotic and prokaryotic cells and had a crucial role in bacterial competition (Ho et al., 2014). T6SS was composed of 13 conserved core genes and few accessory genes which were necessary for function though the genes varied in composition among species (Chang et al., 2014). ImpL, an integral polytopic inner membrane protein, interacted with the essential T6SS component, and ImpK was proved to be necessary for its function of mediating the Hcp secretion in *A. tumefaciens* (Ma et al., 2009). TssL, TssM, and TssJ composed a membrane complex in the T6SS, and played an important role in protein secretion and the assembly of cell surface appendages in *E. coli* (Felisberto-Rodrigues et al., 2011). Notably, the comparative genomic analysis indicated the variation in the gene composition between *Rahnella* strains, but contained the 13 conserved core genes, which might reveal that *Rahnella* perform the normal function of T6SS. To date, T6SS had been proved to be associated with the symbiotic interactions with hosts in many bacteria, but more studies need to explore the function of T6SS in the *Rahnella* strains.

Two-component regulatory system was an essential mechanism for signal transduction, generally consisting of a sensor histidine kinase (HK) and a response regulator (RR) (Lavín et al., 2007). Previous studies showed that GacS/GacA system controlled the biosynthesis of secondary metabolites and extracellular enzymes that were linked with the ecological fitness and tolerance to stress (Heeb and Haas, 2001). PhoB/R TCS played a pivotal role in regulating the inorganic phosphate regulatory system (Yamada et al., 1989). The CheA/CheW TCS regulated chemotaxis and RcsC/RcsB were involved in the motility of bacteria (Majdalani and Gottesman, 2005). Overall, TCS played an essential role in the life activity of bacteria, for the diverse functional TCSs that could adapt to various environments. Recently, it was reported that TCS had a close relationship with biocontrol factors in bacteria, however, the functions of TCSs in ZF458 need to be elucidated.

Selenium (Se) was an important trace element for humans and animals and existed in four states with chemical forms of selenide, elemental selenium, selenite and selenate. Microorganism was reported to play a key role in the biogeochemical cycle of Se in the ecological environment (Skalickova et al., 2017). Many bacteria have been reported to reduce selenate and/or selenite to elemental Se nanoparticles (SeNPs) (Stolz et al., 2006). SeNPs could be used for medicine, therapeutics and environmental remediation due to a variety of advantages including biocompatibility and low toxicity (Wang et al., 2007). Recently, the SeNPs synthesized by bacteria had drawn extensive attention due to their great potential for the bioremediation of polluted environments. *R. aquatilis* HX2 was reported to show a strong tolerance to Se and reduce selenate and selenite to BioSeNPs (Zhu et al., 2018). *R. aquatilis* ZF7 could produce SeNPs from Na_2_SeO_3_, and possessed the most production of SeNPs at 0.85 mM (Yuan et al., 2020). Previous studies proved that the small RNA chaperone Hfq was a key regulator for the reduction of selenium nanoparticles in *R. aquatilis* HX2 (Xu et al., 2020). In this study, many genes linked with selenium metabolism were found in the ZF458 genome, and shared a high homology with those in the genomes of *R. aquatilis* strains ZF7 and HX2, and the results indicated that *R. aceris* ZF458 might synthesize SeNPs and had a widely applications in nano-agriculture.

Acid resistance genes were essential for successful colonization in acidic environments however, most studies on acid resistance genes had been conducted in *E. coli*. For instance, the alternative stress sigma factor (RpoS), was reported to be associated with the oxidative AR system, which allowed *E. coli* O157:H7 to survive in acidic environments (Masuda and Church, 2010). In addition, the glutamate and arginine decarboxylase systems in *E. coli* were also revealed as part of its acid resistance mechanism (Kim et al., 2016). Based on the comparative genomic analysis, many acid resistance genes were observed in the ZF458 genome, but their function still needs to be confirmed.

## Conclusion

**FIGURE 5:**
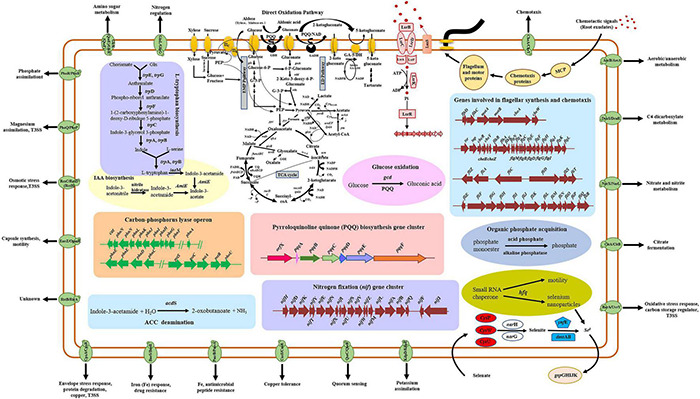
Metabolic pathway map of *R. aceris* ZF458 related to plant growth promotion and environmental adaption.

*Rahnella aceris* ZF458, which was isolated from the swamp soil, exhibited broad-spectrum antagonistic activities against 14 diverse plant pathogens, and displayed a significant effect in controlling *Agrobacterium tumefaciens* on sunflowers. A whole genomic analysis and a phylogenetic analysis showed that strain ZF458 belonged to *R. aceris*, and was closely related to *R. aquatilis*. A comparative genomic analysis indicated that *R. aceris* ZF458 harbored many genes related to nitrogen fixation, IAA production, organic phosphate dissolution, organic acid biosynthesis and PQQ production, which had been proved to be beneficial to plant growth. In addition, large numbers of genes associated with environmental adaption, such as the secretion system, two-component system, selenium metabolism and acid-resistance were found in the ZF458 genome and shared a high homology with *Rahnella* strains ZF7, HX2, Y9602 and ATCC 33071^T^. As far as we know, this was the first study to systematically analyze the genes associated with plant growth promotion and environmental adaption in *R. aceris*. Overall, these features of *R. aceris* ZF458 provided new insight into the comprehensive metabolic pathways (Figure 5), and would assist in studying plant growth promotion and biocontrol application in *R. aceris*.

## Data Availability Statement

The datasets presented in this study can be found in online repositories. The names of the repository/repositories and access ion number(s) can be found below: https://www.ncbi.nlm.nih.gov/bioproject/PRJNA796123.

## Author Contributions

SX, LL, and BL conceived and designed the experiments. SX, YZ, and YP performed the experiments and analyzed the data. SX and LL wrote the manuscript. LL, XX, YS, and AC revised the manuscript. All authors have read and approved of the final version of the manuscript.

## Conflict of Interest

The authors declare that the research was conducted in the absence of any commercial or financial relationships that could be construed as a potential conflict of interest.

## Publisher’s Note

All claims expressed in this article are solely those of the authors and do not necessarily represent those of their affiliated organizations, or those of the publisher, the editors and the reviewers. Any product that may be evaluated in this article, or claim that may be made by its manufacturer, is not guaranteed or endorsed by the publisher.

## Footnotes

1
https://www.pacb.com/support/software-downloads/

2
http://topaz.gatech.edu/GeneMark/

3
http://pfam.xfam.org/

4
http://www.ebi.ac.uk/uniprot/

## Supplementary Material

Supplementary Figure 1 Control efficiency of *R. aceris* ZF458 against *Agrobacterium tumefaciens* on the stem of sunflowers. **(A)** *Agrobacterium tumefaciens* ACCC 19185, **(B)** *Agrobacterium tumefaciens* ACCC 19185 and *R. aceris* ZF458 (V:V = 1:1), **(C)** ZF458, **(D)** control of sterilized water.

Supplementary Figure 2 Production of extracellular enzymes and siderophores by *R. aceris* ZF458. Plate assays for the production of siderophores **(A)** and phosphatases **(B)**.

Supplementary Figure 3 General characteristics of *R. aceris* ZF458. **(A)** Image of ZF458 colony morphology. **(B)** Image of ZF458 cells using transmission electron microscopy (Hitachi 7700, Japan). **(C)** Image of ZF458 cells using scanning electron microscope (Hitachi S3400N, Japan).

Supplementary Figure 4 Growth curve aND pH curve of *R. aceris* ZF458 for different culture times. **(A)** Optical density of ZF458 at different culture times, **(B)** pH of ZF458 at different culture times.

Supplementary Figure 5 Phylogenetic tree highlighting the relative positions of *R. aceris* ZF458 among other *Rahnella* strains. The phylogenetic tree was constructed based on four housekeeping genes (16S rRNA, *gyrB*, *atpD*, and *rpoB*) according to the aligned gene sequences using maximum likelihoods derived from MEGA 6.0 software. Bootstrap values (1,000 replicates) were shown at the branch points. The scale bar indicates 0.05 nucleotide substitutions per nucleotide position.

Supplementary Figure 6 Percentage of the average nucleotide identities (ANI) and *in silico* DNA-DNA hybridization (DDH) among the selected *Rahnella* strains. ANI values were computed for a pairwise genome comparison using the OrthoANIu algorithm. The percentage of ANI was shown on the **top right**. DDH values were calculated by using the Genome-to-Genome Distance Calculator (GGDC). The percentage of DDH was shown on the **bottom left**.

Supplementary Figure 7 Comparisons of Pyrroloquinoline Quinone genes of *R. aceris* ZF458 against four other previously fully sequenced *Rahnella* genomes. The same color represented genes with the same biological function.
