# Supplementary figures and images for: Comparative Genomics Assisted Functional Characterization of Rahnella aceris ZF458 as a Novel Plant Growth Promoting Rhizobacterium
Source: Front Microbiol. 2022 Apr 4;13:850084. doi: 10.3389/fmicb.2022.850084 (PMC9015054; doi:10.3389/fmicb.2022.850084)

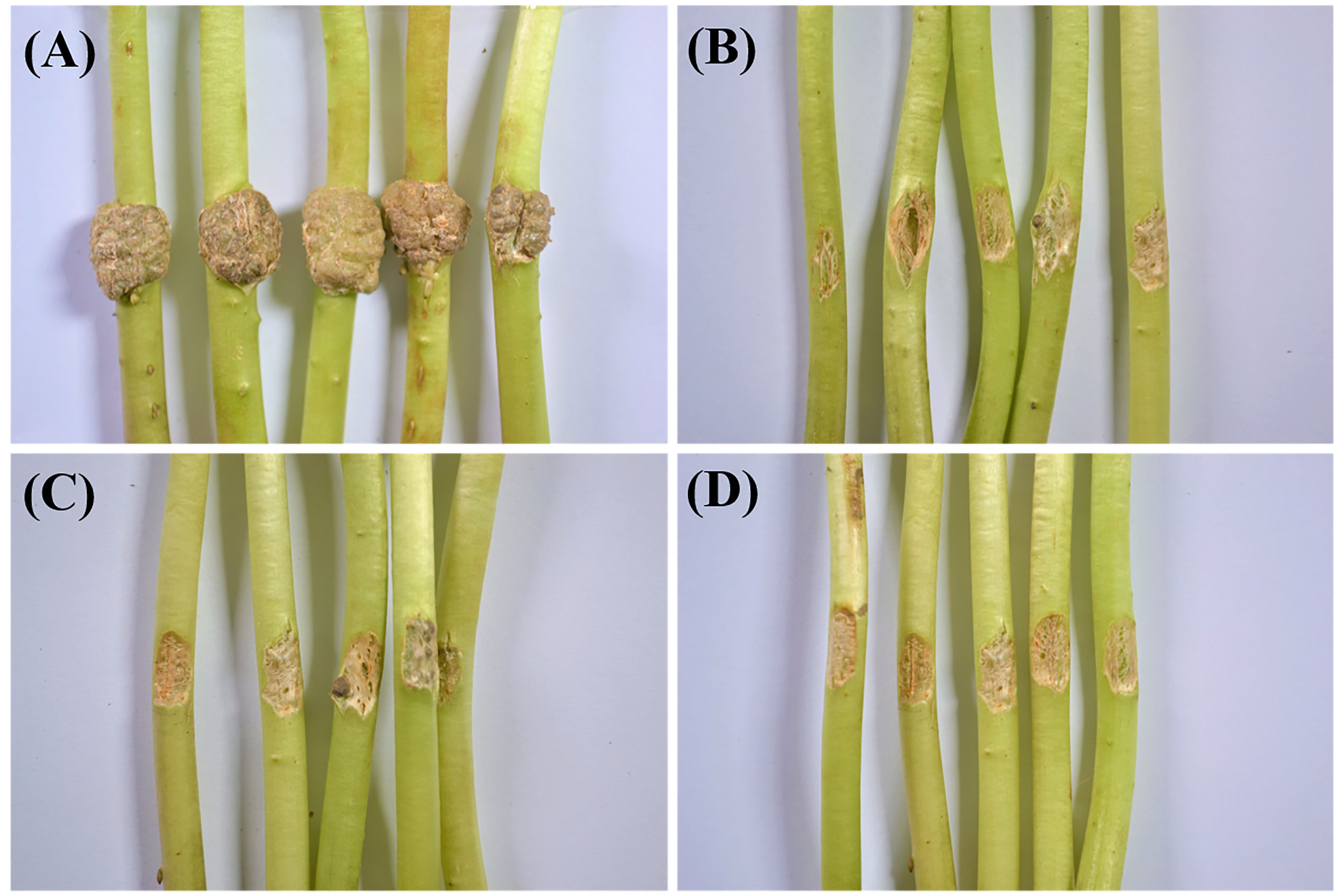

Supplement: Supplementary Figure 1 — Control efficiency of R. aceris ZF458 against Agrobacterium tumefaciens on the stem of sunflowers. (A) Agrobacterium tumefaciens ACCC 19185, (B) Agrobacterium tumefaciens ACCC 19185 and R. aceris ZF458 (V:V = 1:1), (C) ZF458, (D) control of sterilized water. [file Image_1.JPEG]

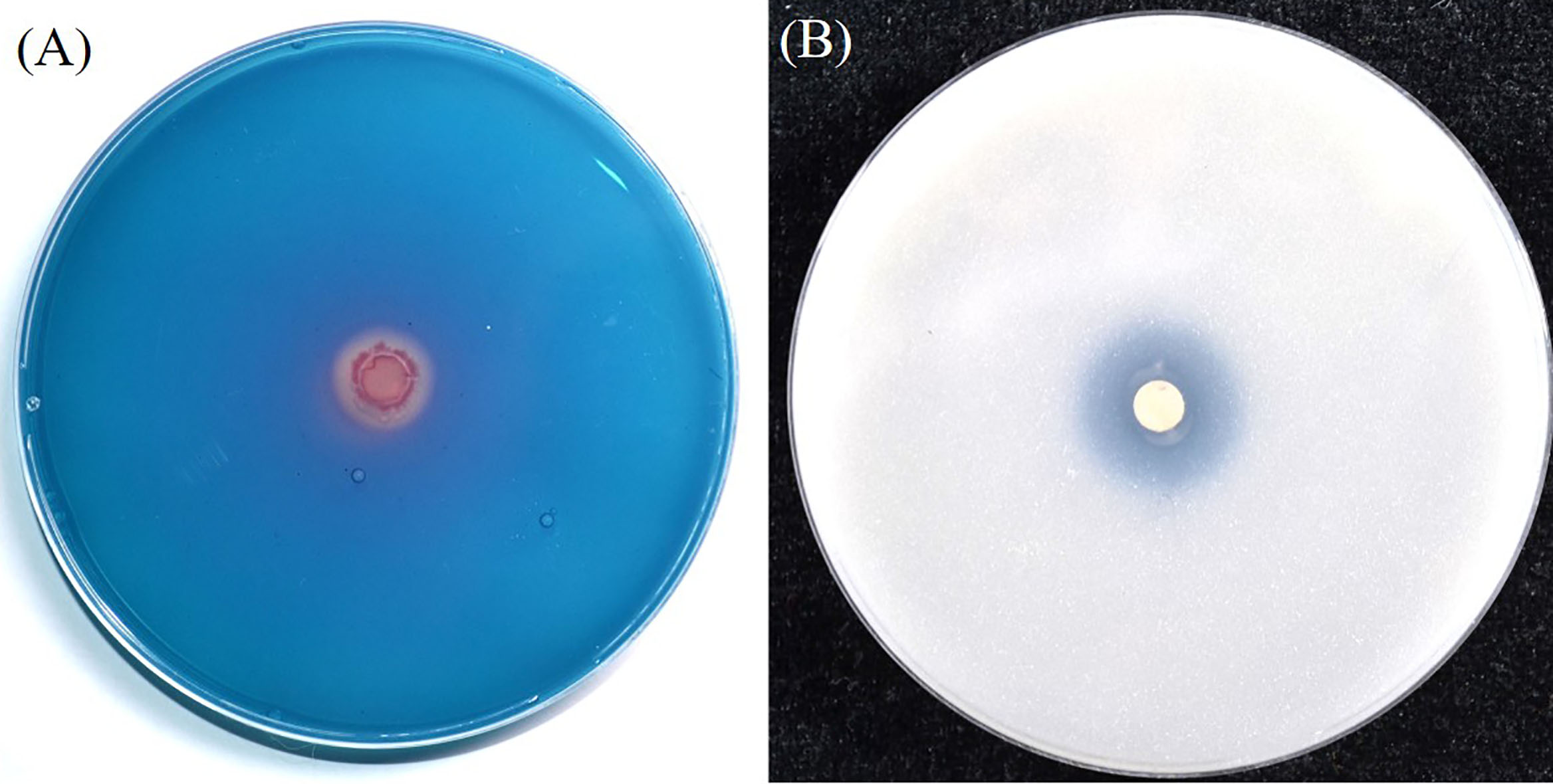

Supplement: Supplementary Figure 2 — Production of extracellular enzymes and siderophores by R. aceris ZF458. Plate assays for the production of siderophores (A) and phosphatases (B). [file Image_2.JPEG]

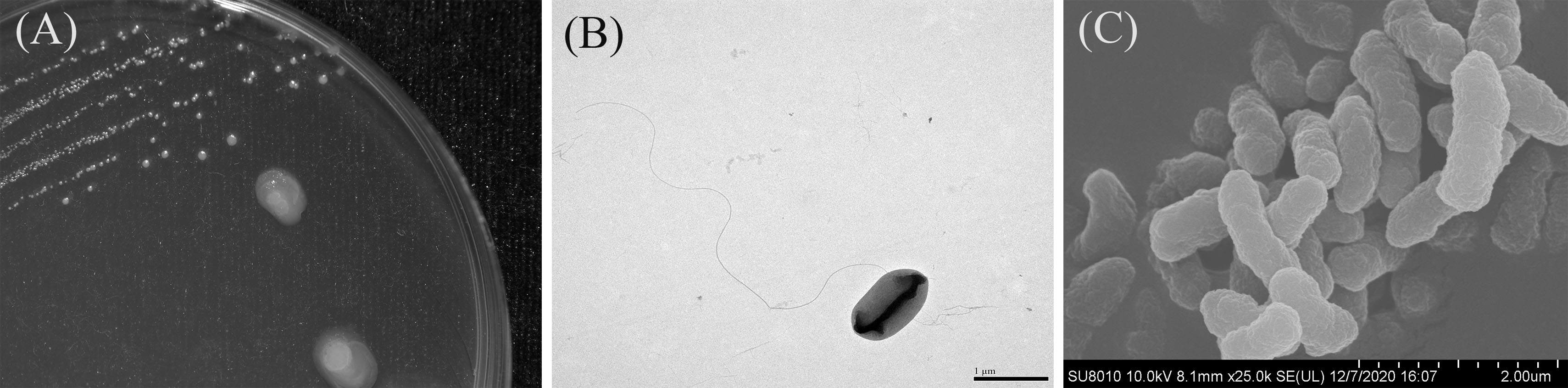

Supplement: Supplementary Figure 3 — General characteristics of R. aceris ZF458. (A) Image of ZF458 colony morphology. (B) Image of ZF458 cells using transmission electron microscopy (Hitachi 7700, Japan). (C) Image of ZF458 cells using scanning electron microscope (Hitachi S3400N, Japan). [file Image_3.JPEG]

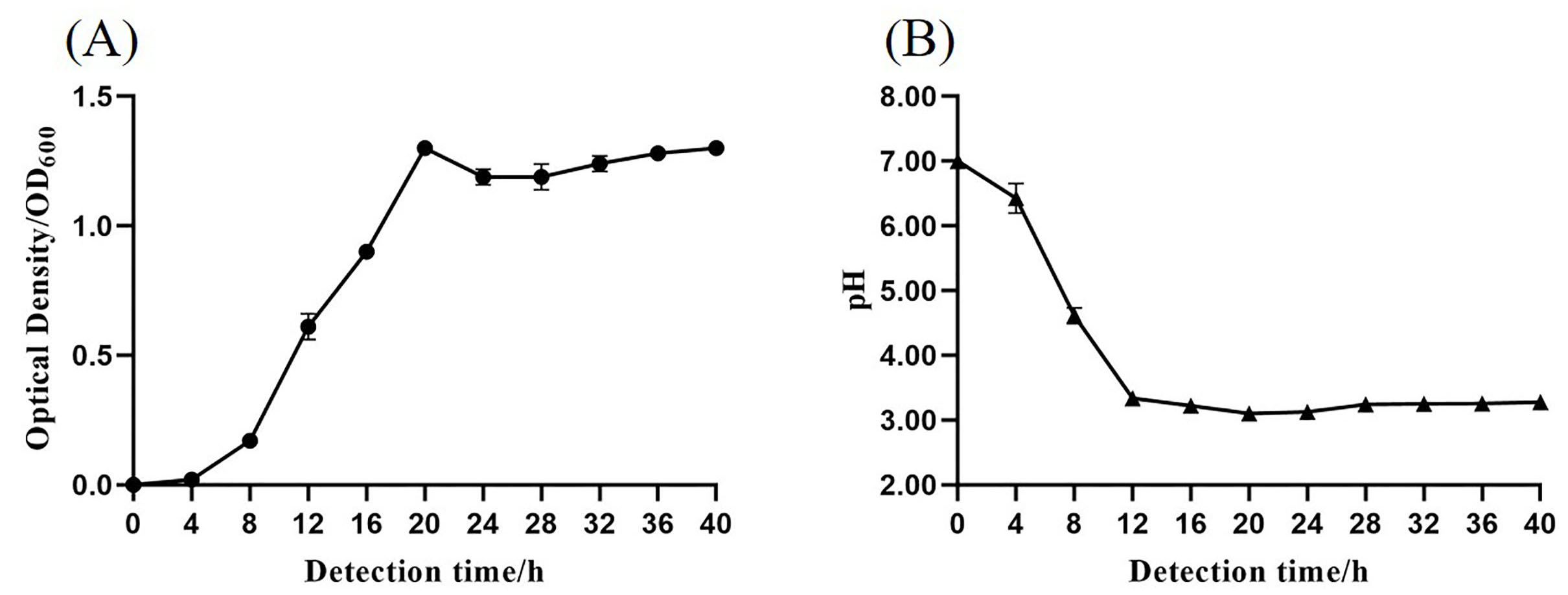

Supplement: Supplementary Figure 4 — Growth curve aND pH curve of R. aceris ZF458 for different culture times. (A) Optical density of ZF458 at different culture times, (B) pH of ZF458 at different culture times. [file Image_4.JPEG]

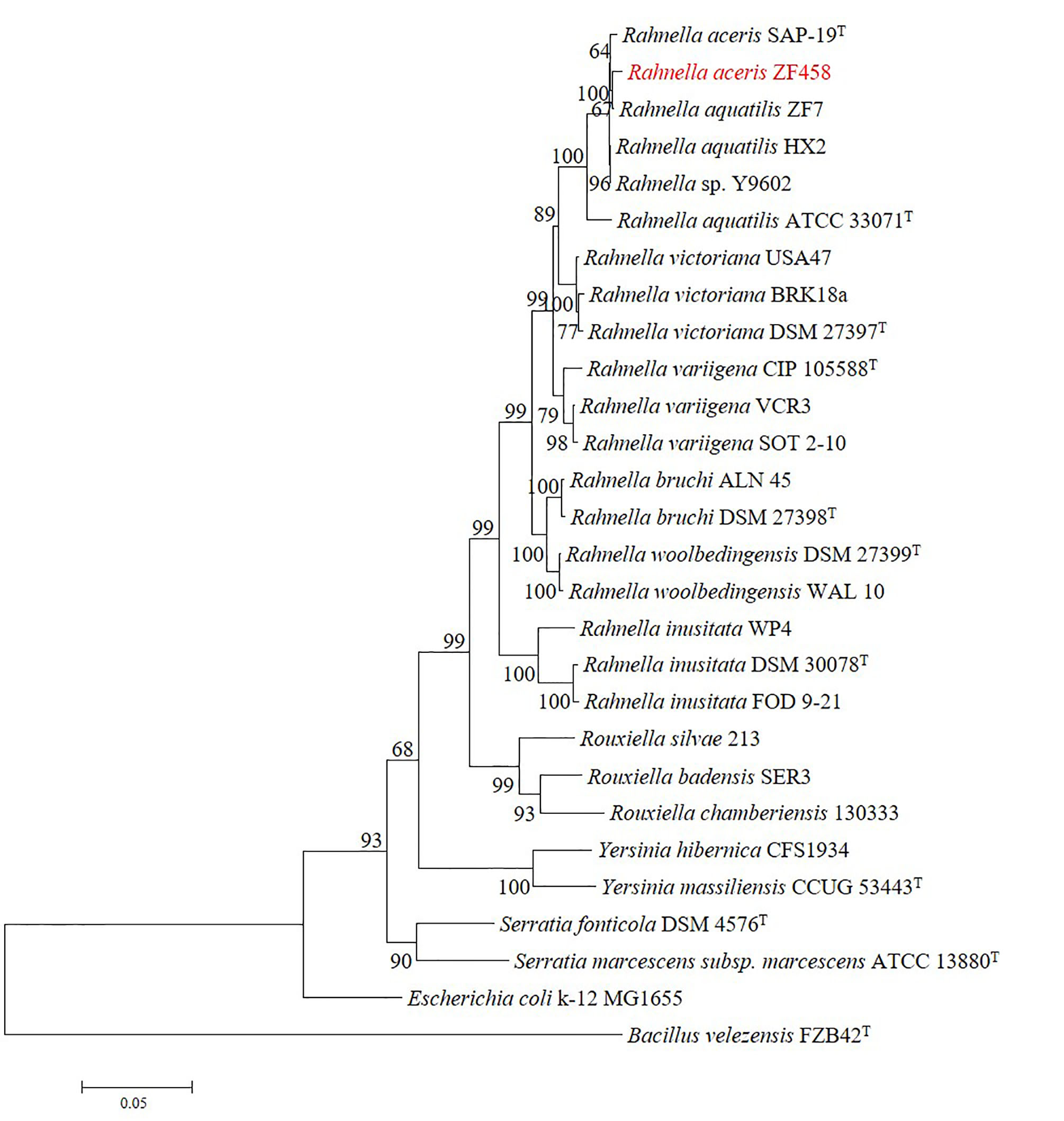

Supplement: Supplementary Figure 5 — Phylogenetic tree highlighting the relative positions of R. aceris ZF458 among other Rahnella strains. The phylogenetic tree was constructed based on four housekeeping genes (16S rRNA, gyrB, atpD, and rpoB) according to the aligned gene sequences using maximum likelihoods derived from MEGA 6.0 software. Bootstrap values (1,000 replicates) were shown at the branch points. The scale bar indicates 0.05 nucleotide substitutions per nucleotide position. [file Image_5.JPEG]

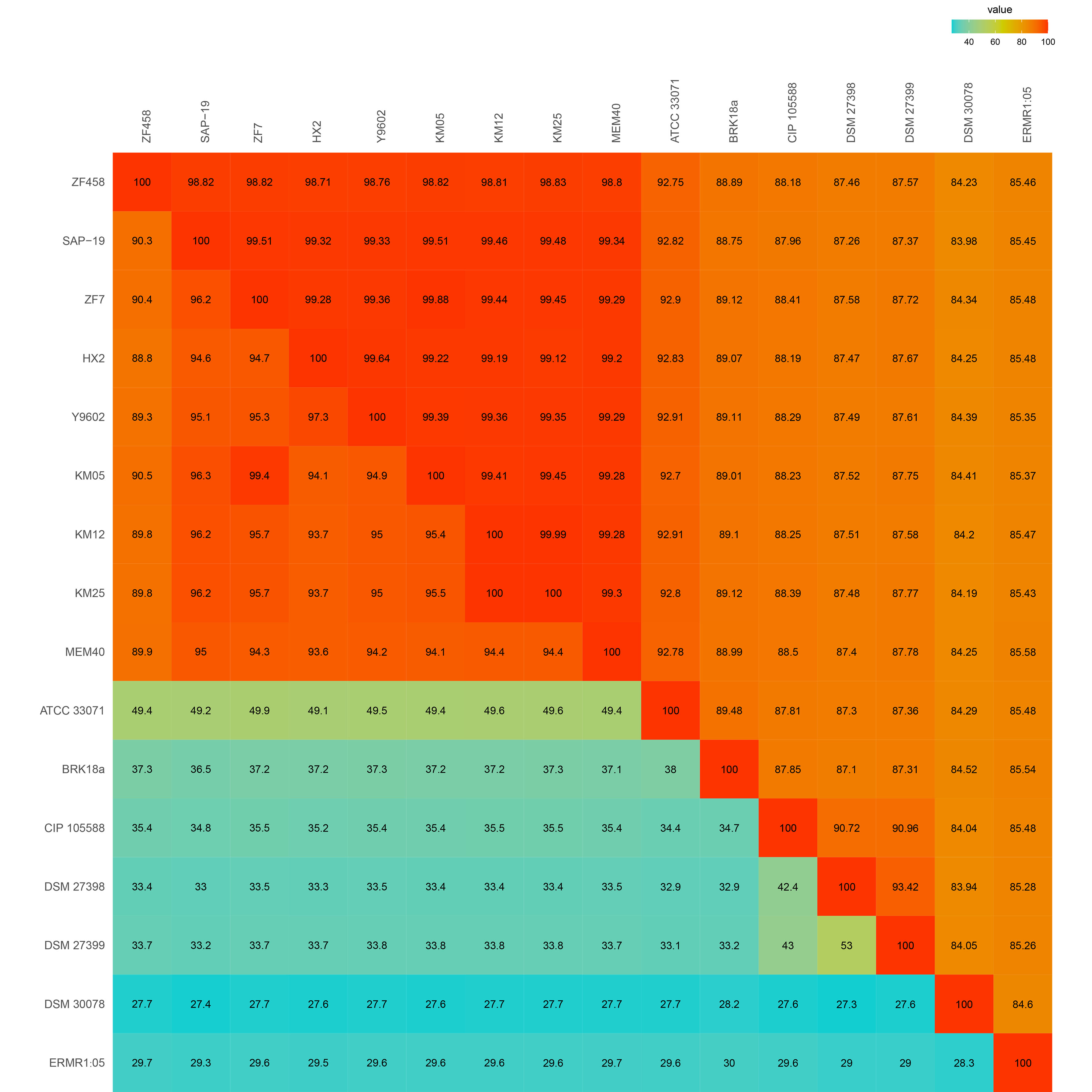

Supplement: Supplementary Figure 6 — Percentage of the average nucleotide identities (ANI) and in silico DNA-DNA hybridization (DDH) among the selected Rahnella strains. ANI values were computed for a pairwise genome comparison using the OrthoANIu algorithm. The percentage of ANI was shown on the top right. DDH values were calculated by using the Genome-to-Genome Distance Calculator (GGDC). The percentage of DDH was shown on the bottom left. [file Image_6.JPEG]

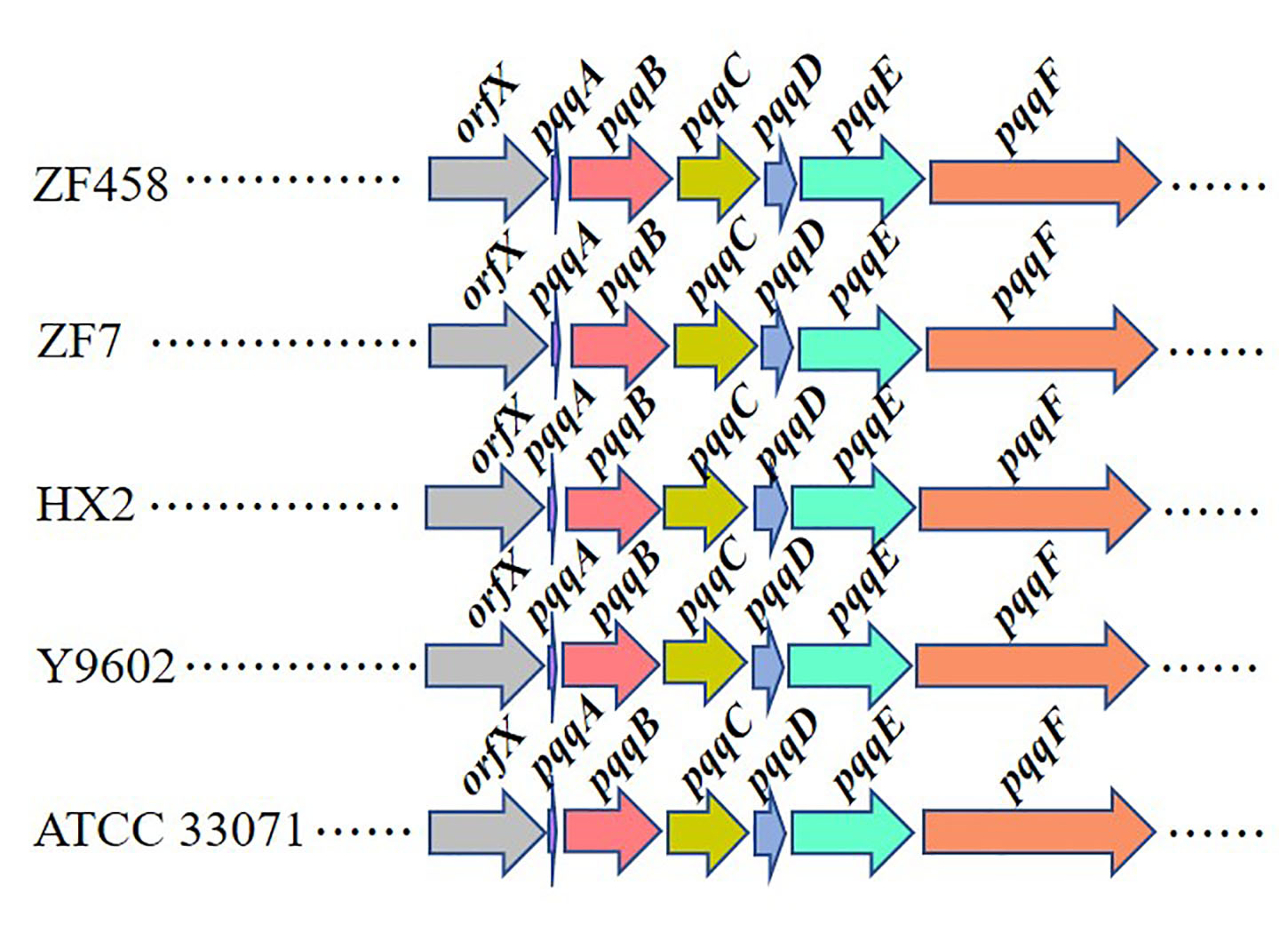

Supplement: Supplementary Figure 7 — Comparisons of Pyrroloquinoline Quinone genes of R. aceris ZF458 against four other previously fully sequenced Rahnella genomes. The same color represented genes with the same biological function. [file Image_7.JPEG]
