# Supplementary material for: Comparative Genomics Assisted Functional Characterization of Rahnella aceris ZF458 as a Novel Plant Growth Promoting Rhizobacterium
Source: Front Microbiol. 2022 Apr 4;13:850084. doi: 10.3389/fmicb.2022.850084 (PMC9015054; doi:10.3389/fmicb.2022.850084)
Supplement: Supplementary file 8 [file Table_1.docx]

**Supplementary Table 1** Locus tag or accession number information of the four housekeeping genes used for the phylogenetic tree construction in this study.

| **Strain (Name)** | **16S rRNA**  **16S ribosomal RNA** | ***gyr*B**  **DNA gyrase subunit B** | ***atp*D**  **ATP synthase subunit beta** | ***rpo*B**  **DNA-directed RNA polymerase subunit Beta** |
| --- | --- | --- | --- | --- |
| *Rahnella aceris* ZF458 | JHW33_RS14930 | JHW33_RS16650 | JHW33_RS16515 | JHW33_RS15015 |
| *Rahnella aceris* SAP-19 | GW591_RS23990 | GW591_RS20000 | GW591_RS20135 | GW591_RS23910 |
| *Rahnella aceris* ZF7 | D3Z09_RS01830 | D3Z09_RS00020 | D3Z09_RS23075 | D3Z09_RS21580 |
| *Rahnella aquatilis* HX2 | Q7S_RS01890 | Q7S_RS00020 | Q7S_RS22375 | Q7S_RS20840 |
| *Rahnella* sp. Y9602 | RAHAQ_RS01880 | RAHAQ_RS00025 | RAHAQ_RS22060 | RAHAQ_RS20535 |
| *Rahnella aquatilis* ATCC 33071 | RAHAQ2_RS01935 | RAHAQ2_RS00025 | RAHAQ2_RS21880 | RAHAQ2_RS21880 |
| *Rahnella victoriana* USA 47 | KF308404 | KF308451 | KF308416 | KF308521 |
| *Rahnella victoriana* BRK18a | C0635_RS11835 | C0635_RS16315 | C0635_RS16455 | C0635_RS18035 |
| *Rahnella victoriana* DSM 27397 | NR_146847.1 | MK391736.1 | MN127833.1 | MN127884.1 |
| *Rahnella variigena* CIP105588 | CKQ54_RS01405 | CKQ54_RS03240 | CKQ54_RS03400 | CKQ54_RS04860 |
| *Rahnella variigena* VCR3 | C5Y41_RS01905 | C5Y41_RS01635 | C5Y41_RS01790 | C5Y41_RS00100 |
| *Rahnella variigena* SOT 2-10 | KF308405 | KF308457 | KF308422 | KF308527 |
| *Rahnella bruchi* ALN45 | KF308408.1 | MK391730.1 | KF308436.1 | KF308541.1 |
| *Rahnella bruchi* DSM 27398 | NR_146845.1 | MN127847.1 | MN127830.1 | MN127881.1 |
| *Rahnella woolbedingensis* 27399 | NR_146848.1 | D6C13_16335 | D6C13_16505 | D6C13_RS23690 |
| *Rahnella woolbedingensis* WAL 10 | KF308410.1 | MK391732.1 | KF308437.1 | KF308542.1 |
| *Rahnella inusitata* WP4 | F4826_RS19115 | F4826_RS18870 | F4826_RS19005 | F4826_RS24235 |
| *Rahnella inusitata* DSM 30078 | NR_146846.1 | KF308464.1 | KF308429.1 | KF308534.1 |
| *Rahnella inusitata* FOD 9-21 | KF308406 | KF308466 | KF308431 | KF308536 |
| *Rouxiella silvae* 213 | NR_156932.1 | BS639_RS16010 | MN127837.1 | KX784900.1 |
| *Rouxiella badensis* SER3 | G3M83_RS00225 | G3M83_RS22105 | G3M83_RS21955 | G3M83_RS20580 |
| *Rouxiella chamberiensis* 130333 | NR_135871.1 | NI27_RS15315 | NI27_RS15450 | KJ526372.1 |
| *Yersinia Hibernica* CFS1934 | D5F51_00090 | D5F51_22070 | D5F51_22250 | D5F51_01550 |
| *Yersinia massiliensis* CCUG 53443 | KJ606910.1 | EF175588.1 | BN68_RS18285 | EF175599.1 |
| *Serratia fonticola* DSM 4576 | WN53_RS01560 | WN53_RS08010 | WN53_RS07790 | WN53_RS09900 |
| *Serritia marcescens subsp. marcescens* ATCC 13880 | FG183_01215 | FG183_00020 | FG183_24445 | FG183_01315 |
| *Escherichia coli* K-12 MG1655 | b3851 | b3699 | b3732 | b3987 |
| *Bacillus velezensis* FZB42 | RBAM_000080 | RBAM_000060 | RBAM_033970 | RBAM_001320 |
