# Supplementary material for: Comparative Genomics Assisted Functional Characterization of Rahnella aceris ZF458 as a Novel Plant Growth Promoting Rhizobacterium
Source: Front Microbiol. 2022 Apr 4;13:850084. doi: 10.3389/fmicb.2022.850084 (PMC9015054; doi:10.3389/fmicb.2022.850084)
Supplement: Supplementary file 9 [file Table_2.DOCX]

**Supplementary Table 2** Genome statistics of *R. aceris* ZF458.

| Attribute | Value | % of total |
| --- | --- | --- |
| Genome size (bp) | 5,602,983 | 100.00 |
| DNA coding (bp) | 4,863,741 | 86.81 |
| DNA G + C (bp) | 2,924,768 | 52.20 |
| Genomics Islands | 159,083 | 2.84 |
| Total genes | 5,248 | 100.00 |
| Protein coding genes | 4,988 | 95.05 |
| RNA genes | 109 | 2.08 |
| Pseudo genes | 88 | 1.68 |
| Genes assigned to KEGGs | 4,954 | 94.40 |
| Genes assigned to SwissProt | 3,316 | 63.19 |
| Genes assigned to COGs | 4,237 | 80.74 |
| Genes assigned to GOs | 3,533 | 67.32 |
| Genes assigned to Pfam | 3,533 | 67.32 |
| Genes assigned to NR | 5,014 | 95.54 |
| Genes assigned to TCDB | 939 | 17.89 |
| Genes assigned to PHI | 672 | 12.80 |
| Genes assigned to VFDB | 470 | 8.96 |
| Genes assigned to Secretory | 342 | 6.52 |
| Genes assigned to CAZY | 210 | 4.00 |
| Genes assigned to T3SS | 226 | 4.31 |
| Genes assigned to CARD | 66 | 1.26 |
| Genes with signal peptides | 428 | 8.16 |
| Genes with transmembrane helices | 1,247 | 23.76 |
| Genes with Secreted Protein | 342 | 6.52 |
| GIs number | 10 | - |
| Prophage_Num | 7 | - |
| CRISPR_Num | N | - |

^a^N/D = not determined
