# Supplementary material for: Comparative Genomics Assisted Functional Characterization of Rahnella aceris ZF458 as a Novel Plant Growth Promoting Rhizobacterium
Source: Front Microbiol. 2022 Apr 4;13:850084. doi: 10.3389/fmicb.2022.850084 (PMC9015054; doi:10.3389/fmicb.2022.850084)
Supplement: Supplementary file 10 [file Table_3.DOCX]

**Supplementary Table 3** Homolog analysis of indole-3-acetic acid biosynthesis genes in *R. aceris* ZF458 and other *Rahnella* strains.

| **Strain** |  | ***Rahnella aceris* ZF458** | | ***R. aquatilis* ZF7** | | ***R. aquatilis* HX2** | | ***Rahnella* sp. Y9602** | | ***R. aquatilis* ATCC 33071** | |
| --- | --- | --- | --- | --- | --- | --- | --- | --- | --- | --- | --- |
| **Genes** | **Product Definition** | **Locus Tag** | **Protein ID** | **Protein ID** | **Homology (%)** | **Protein ID** | **Homology (%)** | **Protein ID** | **Homology (%)** | **Protein ID** | **Homology (%)** |
| *trpG* | glutamine amidotransferase | JHW33_RS19275 | WP_013573858.1 | WP_013573858.1 | 100 | WP_013575925.1 | 100 | WP_013575925.1 | 100 | WP_015697099.1 | 98 |
| *trpE* | Anthranilate synthase component I | JHW33_RS04100 | WP_037034474.1 | WP_013575924.1 | 99 | WP_013575924.1 | 99 | WP_013575924.1 | 99 | WP_015697100.1 | 98 |
| *trpD* | Anthranilate phosphoribosyltransferase | JHW33_RS04090 | WP_037034475.1 | WP_119261742.1 | 99 | WP_013575926.1 | 99 | WP_013575926.1 | 99 | WP_015697098.1 | 98 |
| *trpCF* | Bifunctional indole-3-glycerol phosphate synthase/phosphoribosylanthranilate isomerase | JHW33_RS04085 | WP_200225170.1 | WP_119261743.1 | 99 | WP_013575927.1 | 99 | WP_013575927.1 | 99 | WP_015697097.1 | 93 |
| *trpB* | Tryptophan synthase subunit beta | JHW33_RS04080 | WP_013575928.1 | WP_013575928.1 | 100 | WP_013575928.1 | 100 | WP_013575928.1 | 100 | WP_015697096.1 | 99 |
| *trpA* | Tryptophan synthase alpha chain | JHW33_RS04075 | WP_013575929.1 | WP_013575929.1 | 100 | WP_013575929.1 | 100 | WP_013575929.1 | 100 | WP_015697095.1 | 99 |
| *trpS* | Tryptophanyl-tRNA synthetase | JHW33_RS17965 | WP_037033655.1 | WP_037033655.1 | 100 | WP_013573621.1 | 99 | WP_013573621.1 | 99 | WP_014333546.1 | 99 |
| *trpR* | Trp operon repressor | JHW33_RS13240 | WP_013577084.1 | WP_013577084.1 | 100 | WP_013577084.1 | 100 | WP_013577084.1 | 100 | WP_015698738.1 | 96 |
| *mtr* | Tryptophan permease | JHW33_RS24480 | WP_200227995.1 | WP_013578205.1 | 99 | WP_013578205.1 | 99 | WP_013578205.1 | 96 | WP_014341897.1 | 96 |
| *ipdC* | indolepyruvate decarboxylase | JHW33_RS22375 | WP_015696335.1 | WP_013574453.1 | 99 | WP_013574453.1 | 99 | WP_013574453.1 | 99 | WP_015696334.1 | 97 |
| *acdS* | 1-aminocyclopropane-1-carboxylate deaminase | JHW33_RS05260 | WP_015689960.1 | WP_119261675.1 | 100 | WP_015689960.1 | 100 | WP_041688976.1 | 99 | WP_037039437.1 | 97 |
