# Supplementary material for: Comparative Genomics Assisted Functional Characterization of Rahnella aceris ZF458 as a Novel Plant Growth Promoting Rhizobacterium
Source: Front Microbiol. 2022 Apr 4;13:850084. doi: 10.3389/fmicb.2022.850084 (PMC9015054; doi:10.3389/fmicb.2022.850084)
Supplement: Supplementary file 11 [file Table_4.DOCX]

**Supplementary Table 4** Homolog analysis of phosphate solubilization genes in *R. aceris* ZF458 and other *Rahnella* strains.

| **Strain** |  | ***Rahnella aceris* ZF458** | | ***R. aquatilis* ZF7** | | ***R. aquatilis* HX2** | | ***Rahnella* sp. Y9602** | | ***R. aquatilis* ATCC 33071** | |
| --- | --- | --- | --- | --- | --- | --- | --- | --- | --- | --- | --- |
| **Genes** | **Product Definition** | **Locus Tag** | **Protein ID** | **Protein ID** | **Homology (%)** | **Protein ID** | **Homology (%)** | **Protein ID** | **Homology (%)** | **Protein ID** | **Homology (%)** |
| **organic phosphate acquisition** | | | | | | | | | | | |
| *aphA* | acid phosphatase AphA | JHW33_RS09695 | WP_134705824.1 | WP_119261838.1 | 97 | WP_015690236.1 | 96 | WP_015690236.1 | 96 | NA | NA |
| *phoN* | phosphatase PAP2 family protein | JHW33_RS19375 | WP_013573879.1 | WP_013573879.1 | 100 | WP_013573879.1 | 100 | WP_013573879.1 | 100 | WP_014333784.1 | 94 |
| *iap* | aminopeptidase | JHW33_RS19970 | WP_013574006.1 | WP_013574006.1 | 100 | WP_013574006.1 | 100 | WP_013574006.1 | 100 | WP_015695896.1 | 96 |
| *phoA* | alkaline phosphatase | JHW33_RS06935 | WP_200225998.1 | WP_013575277.1 | 99 | WP_015689871.1 | 99 | WP_013575277.1 | 99 | WP_015697128.1 | 97 |
| *phoB* | phosphate response regulator transcription factor PhoB | JHW33_RS11180 | WP_013576682.1 | WP_013576682.1 | 100 | WP_013576682.1 | 100 | WP_013576682.1 | 100 | WP_013576682.1 | 100 |
| *phoR* | phosphate regulon sensor histidine kinase PhoR | JHW33_RS11175 | WP_037036796.1 | WP_112151727.1 | 99 | WP_013576681.1 | 99 | WP_013576681.1 | 99 | WP_015698354.1 | 98 |
| **carbon-phosphorus lyase** | | | | | | | | | | | |
| *cat* | acetyltransferase | JHW33_RS19175 | WP_013573838.1 | WP_013573838.1 | 100 | WP_014411573.1 | 99 | WP_013573838.1 | 100 | WP_014333722.1 | 95 |
| *phnN* | ribose 1,5-bisphosphokinase | JHW33_RS19180 | WP_014411574.1 | WP_013573839.1 | 99 | WP_014411574.1 | 100 | WP_013573839.1 | 99 | WP_014333723.1 | 94 |
| *phnM* | alpha-D-ribose 1-methylphosphonate 5-triphosphate diphosphatase | JHW33_RS19185 | WP_200223853.1 | WP_013573840.1 | 99 | WP_013573840.1 | 99 | WP_013573840.1 | 99 | WP_014333724.1 | 99 |
| *phnL* | phosphonate C-P lyase system protein PhnL | JHW33_RS19190 | WP_112197547.1 | WP_014411575.1 | 99 | WP_014411575.1 | 99 | WP_013573841.1 | 99 | WP_014333725.1 | 97 |
| *phnK* | phosphonate C-P lyase system protein PhnK | JHW33_RS19195 | WP_013573842.1 | WP_112152052.1 | 100 | WP_014411576.1 | 99 | WP_013573842.1 | 100 | WP_014333726.1 | 97 |
| *phnJ* | alpha-D-ribose 1-methylphosphonate 5-phosphate C-P-lyase PhnJ | JHW33_RS19200 | WP_200223854.1 | WP_013573843.1 | 99 | WP_013573843.1 | 99 | WP_013573843.1 | 99 | WP_014333727.1 | 99 |
| *phnI* | carbon-phosphorus lyase complex subunit PhnI | JHW33_RS19205 | WP_112152053.1 | WP_014333728.1 | 98 | WP_014411577.1 | 99 | WP_013573844.1 | 99 | WP_014333728.1 | 98 |
| *phnH* | phosphonate C-P lyase system protein PhnH | JHW33_RS19210 | WP_037034216.1 | WP_013573845.1 | 99 | WP_013573845.1 | 99 | WP_013573845.1 | 99 | WP_014333729.1 | 97 |
| *phnG* | phosphonate C-P lyase system protein PhnG | JHW33_RS19215 | WP_037034214.1 | WP_013573846.1 | 99 | WP_013573846.1 | 99 | WP_013573846.1 | 99 | WP_014333730.1 | 94 |
| *phnF* | phosphonate metabolism transcriptional regulator PhnF | JHW33_RS19220 | WP_200227267.1 | WP_013573847.1 | 99 | WP_013573847.1 | 99 | WP_013573847.1 | 99 | WP_014333731.1 | 96 |
| *phnA* | alkylphosphonate utilization protein PhnA | JHW33_RS01245 | WP_013574675.1 | WP_013574675.1 | 100 | WP_013574675.1 | 100 | WP_013574675.1 | 100 | WP_015696535.1 | 99 |
| *phnE* | phosphonate ABC transporter, permease protein PhnE | JHW33_RS09530 | WP_013576359.1 | WP_013576359.1 | 100 | WP_013576359.1 | 100 | WP_013576359.1 | 100 | WP_015698039.1 | 99 |
| *phnE* | phosphonate ABC transporter, permease protein PhnE | JHW33_RS09535 | WP_013576360.1 | WP_013576360.1 | 100 | WP_013576360.1 | 100 | WP_013576360.1 | 100 | WP_015698040.1 | 99 |
| *phnD* | phosphonate ABC transporter substrate-binding protein | JHW33_RS09540 | WP_013576361.1 | WP_013576361.1 | 100 | WP_013576361.1 | 100 | WP_013576361.1 | 100 | WP_015698041.1 | 99 |
| *phnC* | phosphonate ABC transporter ATP-binding protein | JHW33_RS09545 | WP_037037171.1 | WP_015690220.1 | 99 | WP_015690220.1 | 99 | WP_013576362.1 | 99 | WP_015698042.1 | 99 |
| *pstS* | phosphate ABC transporter substrate-binding protein PstS | JHW33_RS16540 | WP_013577689.1 | WP_013577689.1 | 100 | WP_013577689.1 | 100 | WP_013577689.1 | 100 | WP_015699284.1 | 99 |
| *pstC* | phosphate ABC transporter permease PstC | JHW33_RS16545 | WP_013577690.1 | WP_013577690.1 | 100 | WP_013577690.1 | 100 | WP_013577690.1 | 100 | WP_015699285.1 | 99 |
| *pstA* | phosphate ABC transporter permease PstA | JHW33_RS16550 | WP_013577691.1 | WP_013577691.1 | 100 | WP_015690614.1 | 99 | WP_013577691.1 | 100 | WP_015699286.1 | 99 |
| *pstB* | phosphate ABC transporter ATP-binding protein PstB | JHW33_RS16555 | WP_013577692.1 | WP_013577692.1 | 100 | WP_013577692.1 | 100 | WP_013577692.1 | 100 | WP_015699287.1 | 100 |
| *phoU* | phosphate signaling complex protein PhoU | JHW33_RS16560 | WP_200223519.1 | WP_013577693.1 | 99 | WP_013577693.1 | 99 | WP_013577693.1 | 99 | WP_015699288.1 | 99 |

NA = not available.
