# Supplementary material for: Comparative Genomics Assisted Functional Characterization of Rahnella aceris ZF458 as a Novel Plant Growth Promoting Rhizobacterium
Source: Front Microbiol. 2022 Apr 4;13:850084. doi: 10.3389/fmicb.2022.850084 (PMC9015054; doi:10.3389/fmicb.2022.850084)
Supplement: Supplementary file 12 [file Table_5.DOCX]

**Supplementary Table 5** Homolog analysis of Pyrroloquinoline Quinone genes in *R. aceris* ZF458 and other *Rahnella* strains.

| **Strain** |  | ***Rahnella aceris* ZF458** | | ***R. aquatilis* ZF7** | | ***R. aquatilis* HX2** | | ***Rahnella* sp. Y9602** | | ***R. aquatilis* ATCC 33071** | |
| --- | --- | --- | --- | --- | --- | --- | --- | --- | --- | --- | --- |
| **Genes** | **Product Definition** | **Locus Tag** | **Protein ID** | **Protein ID** | **Homology (%)** | **Protein ID** | **Homology (%)** | **Protein ID** | **Homology (%)** | **Protein ID** | **Homology (%)** |
| *gcd* | glucose/quinate/shikimate family membrane-bound PQQ-dependent dehydrogenase | JHW33_RS25310 | WP_013578038.1 | WP_112197657.1 | 100 | WP_013578038.1 | 100 | WP_013577979.1 | 100 | WP_014341698.1 | 97 |
| *orfX* | Membrane dipeptidase | JHW33_RS24905 | WP_200227626.1 | WP_119262324.1 | 99 | WP_014416661.1 | 99 | WP_013578121.1 | 99 | WP_014341831.1 | 96 |
| *pqqA (pqqVI)* | pyrroloquinoline quinone precursor peptide PqqA | JHW33_RS24910 | WP_013578120.1 | WP_013578120.1 | 100 | WP_013578120.1 | 100 | WP_013578120.1 | 100 | WP_013578120.1 | 100 |
| *pqqB (pqqV)* | Pyrroloquinoline quinone biosynthesis protein PqqB | JHW33_RS24915 | WP_037032805.1 | WP_013578119.1 | 99 | WP_014416660.1 | 99 | WP_013578119.1 | 99 | WP_014341830.1 | 98 |
| *pqqC (pqqI)* | Pyrroloquinoline quinone biosynthesis protein PqqC | JHW33_RS24920 | WP_037032804.1 | WP_037032804.1 | 100 | WP_037032804.1 | 100 | WP_013578118.1 | 99 | WP_014341829.1 | 96 |
| *pqqD (pqqII)* | Pyrroloquinoline quinone biosynthesis protein PqqD | JHW33_RS24925 | WP_013578117.1 | WP_013578117.1 | 100 | WP_013578117.1 | 100 | WP_013578117.1 | 100 | WP_014341828.1 | 98 |
| *pqqE (pqqIII)* | Pyrroloquinoline quinone biosynthesis protein PqqE | JHW33_RS24930 | WP_200228042.1 | WP_119262325.1 | 99 | WP_049804367.1 | 99 | WP_052300787.1 | 99 | WP_049796161.1 | 99 |
| *pqqF* | Coenzyme PQQ biosynthesis protein PqqF | JHW33_RS24935 | WP_200227628.1 | WP_119262326.1 | 95 | WP_014416657.1 | 95 | WP_013578115.1 | 95 | WP_014341826.1 | 80 |
