# Supplementary material for: Comparative Genomics Assisted Functional Characterization of Rahnella aceris ZF458 as a Novel Plant Growth Promoting Rhizobacterium
Source: Front Microbiol. 2022 Apr 4;13:850084. doi: 10.3389/fmicb.2022.850084 (PMC9015054; doi:10.3389/fmicb.2022.850084)
Supplement: Supplementary file 13 [file Table_6.DOCX]

**Supplementary Table 6** Homolog analysis of bacteria secretion system genes in *R. aceris* ZF458 and other *Rahnella* strains.

| **Strain** |  | ***Rahnella aceris* ZF458** | | ***R. aquatilis* ZF7** | | ***R. aquatilis* HX2** | | ***Rahnella* sp. Y9602** | | ***R. aquatilis* ATCC 33071** | |
| --- | --- | --- | --- | --- | --- | --- | --- | --- | --- | --- | --- |
| **Genes** | **Product Definition** | **Locus Tag** | **Protein ID** | **Protein ID** | **Homology (%)** | **Protein ID** | **Homology (%)** | **Protein ID** | **Homology (%)** | **Protein ID** | **Homology (%)** |
| **Type I secretion** | | | | | | | | | | | |
| *tolC* | outer membrane channel protein TolC | JHW33_RS19760 | WP_013573963.1 | WP_013573963.1 | 100 | WP_015689385.1 | 99 | WP_013573963.1 | 99 | WP_015695859.1 | 99 |
| *raxB* | ABC transporter | JHW33_RS07650 | WP_037035542.1 | WP_013576039.1 | 99 | WP_013576039.1 | 99 | WP_013576039.1 | 99 | WP_015697751.1 | 99 |
| *raxA* | Secretion protein HlyD family protein | JHW33_RS07655 | WP_134706741.1 | WP_112197132.1 | 99 | WP_015690114.1 | 99 | WP_015690114.1 | 99 | WP_037039824.1 | 99 |
| **Type II secretion** | | | | | | | | | | | |
| *pilD* | Prepilin peptidase | JHW33_RS18240 | WP_037035606.1 | WP_119261135.1 | 96 | WP_013573673.1 | 100 | WP_013573673.1 | 100 | WP_014333590.1 | 87 |
| *gspM* | type II secretion system protein M | JHW33_RS18245 | WP_013573674.1 | WP_013573674.1 | 100 | WP_013573674.1 | 100 | WP_013573674.1 | 100 | WP_014333591.1 | 78 |
| *gspL* | type II secretion system protein GspL | JHW33_RS18250 | WP_037035602.1 | WP_014333592.1 | 98 | WP_014333592.1 | 98 | WP_013573675.1 | 98 | WP_014333592.1 | 88 |
| *gspK* | type II secretion system minor pseudopilin GspK | JHW33_RS18255 | WP_037035601.1 | NA |  | WP_013573676.1 | 99 | WP_013573676.1 | 99 | WP_014333593.1 | 93 |
| *gspJ* | type II secretion system minor pseudopilin GspJ | JHW33_RS18260 | WP_037035599.1 | NA | NA | WP_013573677.1 | 97 | WP_013573677.1 | 97 | WP_014333594.1 | 82 |
| *gspI* | type II secretion system minor pseudopilin GspI | JHW33_RS18265 | WP_037035598.1 | NA | NA | WP_013573678.1 | 98 | WP_013573678.1 | 98 | WP_014333595.1 | 86 |
| *gspH* | type II secretion system minor pseudopilin GspH | JHW33_RS18270 | WP_037035596.1 | NA | NA | WP_013573679.1 | 99 | WP_013573679.1 | 99 | WP_014333596.1 | 90 |
| *gspG* | type II secretion system major pseudopilin GspG | JHW33_RS18275 | WP_197060736.1 | NA | NA | WP_173362076.1 | 99 | WP_173362076.1 | 99 | WP_014333597.1 | 96 |
| *gspF* | type II secretion system inner membrane protein GspF | JHW33_RS18280 | WP_014411537.1 | NA | NA | WP_014411537.1 | 100 | WP_013573681.1 | 99 | WP_014333598.1 | 95 |
| *gspE* | type II secretion system ATPase GspE | JHW33_RS18285 | WP_037035592.1 | WP_119262098.1 | 99 | WP_013573682.1 | 99 | WP_013573682.1 | 99 | WP_014333599.1 | 96 |
| *gspD* | type II secretion system secretin GspD | JHW33_RS18290 | WP_037035590.1 | NA | NA | WP_013573683.1 | 99 | WP_013573683.1 | 99 | WP_014333600.1 | 95 |
| *gspC* | type II secretion system protein GspC | JHW33_RS18295 | WP_037035605.1 | NA | NA | WP_013573684.1 | 97 | WP_013573684.1 | 97 | WP_148267105.1 | 81 |
| *gspA* | General secretion pathway protein A | JHW33_RS18300 | WP_200223746.1 | NA | NA | WP_112197971.1 | 98 | WP_112197971.1 | 98 | WP_148267106.1 | 81 |
| **Type III secretion** | | | | | | | | | | | |
| *hrpA* | ATP-dependent RNA helicase HrpA | JHW33_RS06345 | WP_200225535.1 | WP_119261599.1 | 99 | WP_013575405.1 | 99 | WP_013575405.1 | 99 | WP_015697276.1 | 99 |
| *hrpB* | ATP-dependent RNA helicase HrpB | JHW33_RS12515 | WP_200222904.1 | WP_119262087.1 | 99 | WP_013576940.1 | 99 | WP_013576940.1 | 99 | WP_015698604.1 | 95 |
| *radD* | Type III restriction protein res subunit | JHW33_RS01050 | WP_200224955.1 | WP_112198745.1 | 99 | WP_013574636.1 | 99 | WP_013574636.1 | 99 | WP_015696500.1 | 98 |
| **Type IV secretion, T4SS** | | | | | | | | | | | |
| *mltE* | Transglycosylase SLT domain-containing protein | JHW33_RS05845 | WP_200225350.1 | WP_013575507.1 | 99 | WP_013575507.1 | 99 | WP_013575507.1 | 99 | WP_015697351.1 | 99 |
| *virB2* | TrbC/VirB2 family protein | NA | NA | WP_171016405.1 | NA | WP_015689813.1 | NA | NA | NA | WP_037038866.1 | NA |
| *virB3-virB4* | VirB3 family type IV secretion system protein | NA | NA | WP_119261548.1 | NA | WP_015689812.1 | NA | NA | NA | WP_015696807.1 | NA |
| *virB5* | type IV secretion system protein | NA | NA | WP_119261546.1 | NA | WP_015689811.1 | NA | NA | NA | WP_015696802.1 | NA |
| *virB6* | TrbL/VirB6 plasmid conjugal transfer protein | NA | NA | WP_119261547.1 | NA | WP_015689810.1 | NA | NA | NA | WP_015696804.1 | NA |
| *virB7* | Hypothetical protein | NA | NA |  | NA | WP_015689809.1 | NA | NA | NA |  | NA |
| *virB8* | Type IV secretion system protein VirB8 | NA | NA | WP_119262234.1 | NA | WP_015689808.1 | NA | NA | NA | WP_015696802.1 | NA |
| *virB9* | P-type conjugative transfer protein VirB9 | NA | NA | WP_115657482.1 | NA | WP_015689807.1 | NA | NA | NA | WP_015696801.1 | NA |
| *virB10* | Conjugation TrbI-like protein | NA | NA | WP_119261545.1 | NA | WP_015689806.1 | NA | NA | NA | WP_015696800.1 | NA |
| *virB11* | Putative conjugal transfer protein | NA | NA | WP_119261537.1 | NA | WP_015689805.1 | NA | NA | NA | WP_015696799.1 | NA |
| **Type IV secretion, Conjugal transfer region** | | | | | | | | | | | |
| *traA* | conjugal transfer protein | JHW33_RS22415 | WP_200227455.1 | NA | NA | WP_014695870.1 | 99 | NA | NA | NA | NA |
| *traL* | type IV conjugative transfer system protein TraL | JHW33_RS22420 | WP_037035618.1 | NA | NA | WP_041673501.1 | 98 | NA | NA | NA | NA |
| *traE* | type IV conjugative transfer system protein TraE | JHW33_RS22425 | WP_200227458.1 | NA | NA | WP_014695872.1 | 99 | NA | NA | NA | NA |
| *traK* | F-type conjugal transfer protein TraK | JHW33_RS22430 | WP_200227460.1 | NA | NA | WP_014695873.1 | 98 | NA | NA | NA | NA |
| *traB* | Conjugal transfer pilus assembly protein TraB | JHW33_RS22435 | WP_200227462.1 | NA | NA | WP_014695874.1 | 99 | NA | NA | NA | NA |
| *traV* | type IV conjugative transfer system lipoprotein TraV | JHW33_RS22465 | WP_200227476.1 | NA | NA | WP_014695879.1 | 97 | NA | NA | NA | NA |
| *traC* | type IV secretion system protein TraC | JHW33_RS22470 | WP_200227479.1 | NA | NA | WP_014695880.1 | 99 | NA | NA | NA | NA |
| *trbI* | type-F conjugative transfer system protein TrbI | JHW33_RS22475 | WP_200227481.1 | NA | NA | WP_148271893.1 | 99 | NA | NA | NA | NA |
| *traW* | type-F conjugative transfer system protein TraW | JHW33_RS22480 | WP_121019097.1 | NA | NA | WP_014695882.1 | 99 | NA | NA | NA | NA |
| *traU* | conjugal transfer pilus assembly protein TraU | JHW33_RS22505 | WP_200227489.1 | NA | NA | WP_014695886.1 | 99 | NA | NA | NA | NA |
| *trbC* | type-F conjugative transfer system pilin assembly protein TrbC | JHW33_RS22510 | JHW33_RS22510 | NA | NA | WP_014695887.1 | 99 | NA | NA | NA | NA |
| *traN* | type-F conjugative transfer system mating-pair stabilization protein TraN | JHW33_RS22515 | WP_200227493.1 | NA | NA | WP_014695888.1 | 99 | NA | NA | NA | NA |
| *traF* | type-F conjugative transfer system pilin assembly protein TraF | JHW33_RS22520 | WP_200227567.1 | NA | NA | WP_014695889.1 | 98 | NA | NA | NA | NA |
| *trbB* | F-type conjugal transfer protein TrbB | JHW33_RS22540 | WP_200227504.1 | NA | NA | WP_049804374.1 | 99 | NA | NA | NA | NA |
| *traH* | conjugal transfer protein TraH | JHW33_RS22550 | WP_014695895.1 | NA | NA | WP_014695895.1 | 100 | NA | NA | NA | NA |
| *traG* | Conjugal transfer mating pair stabilization protein TraG | JHW33_RS22555 | WP_200227508.1 | NA | NA | WP_014695896.1 | 87 | NA | NA | NA | NA |
| *traT* | complement resistance protein TraT | JHW33_RS22565 | WP_200227512.1 | NA | NA | WP_014695898.1 | 99 | NA | NA | NA | NA |
| *traD* | type IV conjugative transfer system coupling protein TraD | JHW33_RS22585 | WP_200227518.1 | NA | NA | WP_014695900.1 | 94 | NA | NA | NA | NA |
| *traI* | Conjugal transfer nickase/helicase TraI | JHW33_RS22590 | WP_200227520.1 | NA | NA | WP_014695901.1 | 89 | NA | NA | NA | NA |
| **Type VI secretion** | | | | | | | | | | | |
| *hcp* | Type VI secretion system effector | JHW33_RS15330 | WP_013577460.1 | WP_037033840.1 | 100 | WP_013575863.1 | 100 | WP_013575863.1 | 100 | WP_014333353.1 | 83 |
| *hcp* | hydroxylamine reductase | JHW33_RS01635 | WP_013574764.1 | WP_112198010.1 | 100 | WP_013574764.1 | 100 | WP_013574764.1 | 100 | WP_015696597.1 | 98 |
| *vgrG* | type VI secretion system tip protein VgrG | JHW33_RS07665 | WP_200226483.1 | NA | NA | WP_013576042.1 | 97 | WP_013576042.1 | 97 | WP_014333418.1 | 94 |
| *vgrG* | type VI secretion system tip protein VgrG | JHW33_RS13845 | WP_200223004.1 | NA | NA | WP_015690502.1 | 97 | WP_013577181.1 | 97 | WP_014333763.1 | 95 |
| *vgrG* | type VI secretion system tip protein VgrG | JHW33_RS17255 | WP_200223630.1 | NA | NA | NA | NA | NA | NA | WP_015697754.1 | 96 |
| *hcp* | Type VI secretion system effector | NA | NA | ND | ND | WP_013576956.1 | NA | WP_013576956.1 | NA | WP_015696031.1 | NA |
| *hcp* | type VI secretion system tube protein Hcp | NA | NA | NA | NA | WP_013577460.1 | NA | WP_013577460.1 | NA | WP_015697956.1 | NA |
| *hcp* | type VI secretion system tube protein Hcp | NA | NA | NA | NA | WP_013577483.1 | NA | WP_013577483.1 | NA | NA | NA |
| *tssB* | type VI secretion system contractile sheath small subunit | JHW33_RS24640 | WP_013578173.1 | WP_119262431.1 | 100 | WP_013577825.1 | 100 | WP_013577825.1 | 100 | WP_014341866.1 | 96 |
| *tssC* | type VI secretion system contractile sheath large subunit | JHW33_RS23840 | WP_037033056.1 | WP_119262430.1 | 99 | WP_014416544.1 | 99 | WP_014416544.1 | 99 | WP_037040958.1 | 99 |
| *tssK* | type VI secretion system baseplate subunit TssK | JHW33_RS24585 | WP_013578184.1 | WP_013578184.1 | 100 | WP_013578184.1 | 100 | WP_013578184.1 | 100 | WP_014341877.1 | 95 |
| *tssL* | type VI secretion system protein TssL, short form | NA | NA | WP_119262428.1 | NA | WP_013577828.1 | NA | WP_013577828.1 | NA | WP_014341984.1 | NA |
| *tssH* | type VI secretion system ATPase TssH | JHW33_RS24570 | WP_200228006.1 | WP_119262301.1 | 99 | WP_014416693.1 | 99 | WP_013578187.1 | 99 | WP_014341880.1 | 95 |
| *tssI* | type VI secretion system tip protein VgrG | JHW33_RS24610 | WP_037032869.1 | WP_119262416.1 | 99 | WP_014416689.1 | 99 | WP_013578179.1 | 99 | WP_014341872.1 | 96 |
| *vasK* | type VI secretion protein VasK | NA | NA | WP_119262418.1 | NA | WP_013577840.1 | NA | WP_013577840.1 | NA | WP_014341605.1 | NA |
| *tssF* | type VI secretion system baseplate subunit TssF | JHW33_RS24620 | WP_200228014.1 | WP_013578177.1 | 99 | WP_013578177.1 | 99 | WP_013578059.1 | 99 | WP_014341606.1 | 95 |
| *tssG* | type VI secretion system baseplate subunit TssG | JHW33_RS24615 | WP_013578178.1 | WP_013578178.1 | 100 | WP_013578178.1 | 100 | WP_013578178.1 | 100 | WP_014341871.1 | 93 |
| *tssJ* | type VI secretion lipoprotein TssJ | JHW33_RS24590 | WP_037032877.1 | WP_119262303.1 | 99 | WP_013578183.1 | 99 | WP_013578183.1 | 99 | WP_049796162.1 | 93 |
| *tssA* | type VI secretion system protein TssA | JHW33_RS23805 | WP_200227857.1 | WP_119262417.1 | 99 | WP_013577849.1 | 99 | WP_013577849.1 | 99 | WP_014341614.1 | 98 |
| *tssE* | Type VI secretion system lysozyme-related protein | JHW33_RS23800 | WP_134705236.1 | WP_037033085.1 | 93 | WP_013577850.1 | 93 | WP_013577850.1 | 93 | WP_014341615.1 | 99 |
| *vasL* | type VI secretion system ImpA family N-terminal domain-containing protein | JHW33_RS23790 | WP_200227855.1 | WP_119262412.1 | 98 | WP_013577853.1 | 98 | WP_013577853.1 | 98 | WP_014341617.1 | 93 |
| *hcp* | Hcp family type VI secretion system effector | JHW33_RS23575 | WP_013577896.1 | WP_013578023.1 | 100 | WP_013577896.1 | 100 | WP_013577896.1 | 100 | WP_014341647.1 | 99 |
| *tssB* | type VI secretion system contractile sheath small subunit | JHW33_RS24640 | WP_013578173.1 | WP_013578173.1 | 100 | WP_013578173.1 | 100 | WP_013578173.1 | 100 | WP_014341981.1 | 96 |
| *tssC* | type VI secretion system contractile sheath large subunit | JHW33_RS24635 | WP_013578174.1 | WP_119262304.1 | 100 | WP_013578174.1 | 100 | WP_013578174.1 | 100 | WP_014341867.1 | 98 |
| *hcp* | type VI secretion system tube protein Hcp | JHW33_RS24630 | WP_013578175.1 | WP_013578175.1 | 100 | WP_013578175.1 | 100 | WP_013578175.1 | 100 | WP_014341942.1 | 54 |
| *impF* | GPW/gp25 family protein | JHW33_RS24625 | WP_013578176.1 | WP_013578176.1 | 100 | WP_014416688.1 | 100 | WP_013578176.1 | 99 | WP_014341869.1 | 96 |
| *tssG* | type VI secretion system baseplate subunit TssG | JHW33_RS24615 | WP_013578178.1 | WP_013578178.1 | 100 | WP_013578178.1 | 100 | WP_013578178.1 | 100 | WP_014341871.1 | 93 |
| *tssA* | type VI secretion system protein TssA | JHW33_RS24595 | WP_200228010.1 | WP_013578182.1 | 99 | WP_013578182.1 | 99 | WP_013578182.1 | 99 | WP_014341875.1 | 92 |
| *impK* | DotU family type IV/VI secretion system protein | JHW33_RS24580 | WP_013578185.1 | WP_112197334.1 | 100 | WP_013578185.1 | 100 | WP_013578185.1 | 100 | WP_014341878.1 | 90 |
| *impL* | Hypothetical protein | JHW33_RS24575 | WP_200228008.1 | WP_119262302.1 | 99 | WP_014416692.1 | 99 | WP_013578186.1 | 99 | WP_014341879.1 | 89 |
| **Twin arginine targeting** | | | | | | | | | | | |
| *tatE* | twin-arginine translocase subunit TatE | JHW33_RS10290 | WP_013576504.1 | WP_013576504.1 | 100 | WP_013577433.1 | 100 | WP_013576504.1 | 100 | WP_013577433.1 | 100 |
| *tatB* | Sec-independent protein translocase protein TatB | JHW33_RS15175 | WP_013577432.1 | WP_013577432.1 | 100 | WP_013577432.1 | 100 | WP_013577432.1 | 100 | WP_015699057.1 | 90 |
| *tatC* | Sec-independent protein translocase subunit TatC | JHW33_RS15170 | WP_013577431.1 | WP_013577431.1 | 100 | WP_013577431.1 | 100 | WP_013577431.1 | 100 | WP_013577431.1 | 100 |
| *tatE* | twin-arginine translocase subunit TatE | JHW33_RS15180 | WP_013577433.1 | WP_013577433.1 | 100 | WP_013576504.1 | 100 | WP_013577433.1 | 100 | WP_013576504.1 | 100 |
| **Sec (secretion) system** | | | | | | | | | | | |
| *secY* | Preprotein translocase subunit SecY | JHW33_RS18415 | WP_013573703.1 | WP_013573703.1 | 100 | WP_013573703.1 | 100 | WP_013573703.1 | 100 | WP_014333605.1 | 100 |
| *secG* | Preprotein translocase subunit SecG | JHW33_RS19055 | WP_013573818.1 | WP_119261167.1 | 99 | WP_014411569.1 | 95 | WP_013573818.1 | 100 | WP_014333700.1 | 98 |
| *secF* | Preprotein translocase subunit SecF | JHW33_RS11110 | WP_013576668.1 | WP_013576668.1 | 100 | WP_013576668.1 | 100 | WP_013576668.1 | 100 | WP_015698343.1 | 80 |
| *secD* | Preprotein translocase subunit SecD | JHW33_RS11115 | WP_015690312.1 | WP_015690312.1 | 100 | WP_015690312.1 | 100 | WP_015690312.1 | 100 | WP_037040139.1 | 95 |
| *yajC* | Preprotein translocase subunit YajC | JHW33_RS11120 | WP_013576670.1 | WP_013576670.1 | 100 | WP_013576670.1 | 100 | WP_013576670.1 | 100 | WP_013576670.1 | 100 |
| *secA* | Preprotein translocase subunit SecA | JHW33_RS12810 | WP_013577001.1 | WP_119262102.1 | 100 | WP_013577001.1 | 100 | WP_013577001.1 | 100 | WP_015698662.1 | 99 |
| *secM* | SecA regulator SecM | JHW33_RS12815 | WP_037033422.1 | WP_037033422.1 | 99 | WP_013577002.1 | 99 | WP_013577002.1 | 99 | WP_015698663.1 | 98 |
| *secE* | Preprotein translocase subunit SecE | JHW33_RS15045 | WP_013577415.1 | WP_013577415.1 | 100 | WP_013577415.1 | 100 | WP_013577415.1 | 100 | WP_013577415.1 | 100 |
| *secB* | Preprotein translocase subunit SecB | JHW33_RS16065 | WP_013577602.1 | WP_013577602.1 | 100 | WP_013577602.1 | 100 | WP_013577602.1 | 100 | WP_013577602.1 | 100 |
| *yidC* | Membrane protein insertase | JHW33_RS16615 | WP_013577704.1 | WP_013577704.1 | 100 | WP_013577704.1 | 100 | WP_013577704.1 | 100 | WP_015699299.1 | 99 |
| **SRP components** | | | | | | | | | | | |
| *ffh* | Signal recognition particle protein | JHW33_RS20130 | WP_200224028.1 | WP_013574034.1 | 99 | WP_013574034.1 | 99 | WP_013574034.1 | 99 | WP_015695928.1 | 99 |
| *ftsY* | Signal recognition particle-docking protein FtsY | JHW33_RS15395 | WP_153376742.1 | WP_119262174.1 | 99 | WP_013577473.1 | 99 | WP_013577473.1 | 99 | WP_015699089.1 | 90 |

ND = not determined; NA = not available.
