# Supplementary material for: Comparative Genomics Assisted Functional Characterization of Rahnella aceris ZF458 as a Novel Plant Growth Promoting Rhizobacterium
Source: Front Microbiol. 2022 Apr 4;13:850084. doi: 10.3389/fmicb.2022.850084 (PMC9015054; doi:10.3389/fmicb.2022.850084)
Supplement: Supplementary file 14 [file Table_7.DOCX]

**Supplementary Table 7** Genes associated with the two-component system in *R. aceris* ZF458 and other *Rahnella* strains.

| **Strain** |  | ***Rahnella aceris* ZF458** | | ***R. aquatilis* ZF7** | | ***R. aquatilis* HX2** | | ***Rahnella* sp. Y9602** | | ***R. aquatilis* ATCC 33071** | |
| --- | --- | --- | --- | --- | --- | --- | --- | --- | --- | --- | --- |
| **Genes** | **Product Definition** | **Locus Tag** | **Protein ID** | **Protein ID** | **Homology (%)** | **Protein ID** | **Homology (%)** | **Protein ID** | **Homology (%)** | **Protein ID** | **Homology (%)** |
| *PhoR* | phosphate regulon sensor histidine kinase PhoR | JHW33_RS11175 | WP_037036796.1 | WP_112151727.1 | 99 | WP_013576681.1 | 99 | WP_013576681.1 | 99 | WP_015698354.1 | 98 |
| *PhoB* | phosphate regulon response regulator PhoB | JHW33_RS11180 | WP_013576682.1 | WP_013576682.1 | 100 | WP_013576682.1 | 100 | WP_013576682.1 | 100 | WP_013576682.1 | 100 |
| *PhoQ* | sensor histidine kinase PhoQ | JHW33_RS08840 | WP_013576219.1 | WP_013576219.1 | 100 | WP_013576219.1 | 100 | WP_013576219.1 | 100 | WP_015697910.1 | 99 |
| *PhoP* | response regulator PhoP | JHW33_RS08835 | WP_013576218.1 | WP_013576218.1 | 100 | WP_013576218.1 | 100 | WP_013576218.1 | 100 | WP_013576218.1 | 100 |
| *EnvZ* | osmolarity sensor histidine kinase EnvZ | JHW33_RS17865 | WP_013573600.1 | WP_013573600.1 | 100 | WP_013573600.1 | 100 | WP_013573600.1 | 100 | WP_014333528.1 | 99 |
| *OmpR* | phosphate regulon response regulator OmpR | JHW33_RS17860 | WP_009635740.1 | WP_119261115.1 | 100 | WP_009635740.1 | 100 | WP_009635740.1 | 100 | WP_009635740.1 | 100 |
| *RstB* | sensor histidine kinase RstB | JHW33_RS06325 | WP_013575409.1 | WP_013575409.1 | 100 | WP_013575409.1 | 100 | WP_013575409.1 | 100 | WP_015697482.1 | 98 |
| *RstA* | response regulator RstA | JHW33_RS06320 | WP_013575410.1 | WP_013575410.1 | 100 | WP_013575410.1 | 100 | WP_013575410.1 | 100 | WP_015697481.1 | 98 |
| *CpxA* | sensor histidine kinase CpxA | JHW33_RS16045 | WP_037035328.1 | WP_119262198.1 | 99 | WP_013577598.1 | 99 | WP_013577598.1 | 99 | WP_015699199.1 | 99 |
| *CpxR* | response regulator CpxR | JHW33_RS16040 | WP_013577597.1 | WP_013577597.1 | 100 | WP_013577597.1 | 100 | WP_013577597.1 | 99 | WP_015699198.1 | 99 |
| *BaeS* | sensor histidine kinase BaeS | JHW33_RS22115 | WP_200224439.1 | WP_112151270.1 | 99 | WP_013574408.1 | 99 | WP_013574408.1 | 99 | WP_015696295.1 | 99 |
| *BaeR* | response regulator BaeR | JHW33_RS22110 | WP_037036367.1 | WP_037036367.1 | 100 | WP_013574407.1 | 99 | WP_013574407.1 | 99 | WP_015696294.1 | 97 |
| *PmrB* | sensor histidine kinase BasS | JHW33_RS19010 | WP_200223845.1 | WP_112197543.1 | 100 | WP_013573809.1 | 99 | WP_013573809.1 | 99 | WP_014333692.1 | 91 |
| *PmrA* | response regulator BasR | JHW33_RS19015 | WP_013573810.1 | WP_013573810.1 | 100 | WP_013573810.1 | 100 | WP_013573810.1 | 100 | WP_014333693.1 | 98 |
| *CusS* | heavy metal sensor histidine kinase CusS | JHW33_RS01360 | WP_200224971.1 | WP_119261414.1 | 99 | WP_015689552.1 | 99 | WP_052300778.1 | 99 | WP_015696551.1 | 95 |
| *CusR* | copper resistance phosphate regulon response regulator CusR | JHW33_RS01355 | WP_013574706.1 | WP_013574706.1 | 100 | WP_013574706.1 | 100 | WP_013574706.1 | 100 | WP_015696552.1 | 99 |
| *QseC* | sensor histidine kinase QseC | JHW33_RS12550 | WP_200227233.1 | WP_173362117.1 | 99 | WP_173362117.1 | 99 | WP_173362117.1 | 99 | WP_193785529.1 | 94 |
| *QseB* | response regulator QseB | JHW33_RS12555 | WP_121019716.1 | WP_013576948.1 | 99 | WP_013576948.1 | 99 | WP_013576948.1 | 99 | WP_015698611.1 | 96 |
| *CheA* | sensor kinase CheA | JHW33_RS03140 | WP_153375938.1 | WP_153375938.1 | 100 | WP_153375938.1 | 100 | WP_153375938.1 | 100 | WP_193785491.1 | 98 |
| *CheY* | chemotaxis protein CheY | JHW33_RS03440 | WP_013575154.1 | WP_013575154.1 | 100 | WP_013575154.1 | 100 | WP_013575154.1 | 100 | WP_013575154.1 | 100 |
| *KdpD* | sensor histidine kinase KdpD | JHW33_RS10045 | WP_013576460.1 | WP_037037073.1 | 100 | WP_013576460.1 | 100 | WP_013576460.1 | 100 | WP_015698132.1 | 98 |
| *KdpE* | KDP operon response regulator KdpE | JHW33_RS10050 | WP_013576461.1 | WP_013576461.1 | 100 | WP_013576461.1 | 100 | WP_013576461.1 | 100 | WP_015698133.1 | 97 |
| *ArcB* | aerobic respiration control sensor histidine kinase ArcB | JHW33_RS14105 | WP_013577233.1 | WP_112198094.1 | 100 | WP_015690518.1 | 100 | WP_013577233.1 | 99 | WP_015698876.1 | 99 |
| *ArcA* | aerobic respiration control protein ArcA | JHW33_RS13215 | WP_013577079.1 | WP_013577079.1 | 100 | WP_013577079.1 | 100 | WP_013577079.1 | 100 | WP_013577079.1 | 100 |
| *NarX* | nitrate/nitrite sensor histidine kinase NarX | JHW33_RS06285 | WP_131637636.1 | WP_013575417.1 | 99 | WP_015689909.1 | 99 | WP_013575417.1 | 99 | WP_015697474.1 | 96 |
| *NarL* | nitrate/nitrite response regulator NarL | JHW33_RS06290 | WP_013575416.1 | WP_013575416.1 | 100 | WP_013575416.1 | 100 | WP_013575416.1 | 100 | WP_015697475.1 | 99 |
| *RcsC* | capsular synthesis sensor histidine kinase RcsC | JHW33_RS00965 | WP_037036178.1 | WP_013574619.1 | 99 | WP_015689531.1 | 99 | WP_013574619.1 | 99 | WP_015696485.1 | 97 |
| *RcsD* | sensor histidine kinase RcsD | JHW33_RS00975 | WP_037036176.1 | WP_013574621.1 | 99 | WP_013574621.1 | 99 | WP_013574621.1 | 99 | WP_015696487.1 | 96 |
| *RcsB* | captular synthesis response regulator RcsB | JHW33_RS00970 | WP_013574620.1 | WP_013574620.1 | 100 | WP_013574620.1 | 100 | WP_013574620.1 | 100 | WP_013574620.1 | 100 |
| *UvrY* | invasion response regulator UvrY | JHW33_RS09215 | WP_013576296.1 | WP_013576296.1 | 100 | WP_013576296.1 | 99 | WP_013576296.1 | 99 | WP_015697981.1 | 92 |
| *CitA* | cit operon sensor histidine kinase CitA | JHW33_RS05375 | WP_200225245.1 | WP_112152452.1 | 99 | WP_013574090.1 | 99 | WP_013574090.1 | 99 | WP_015695980.1 | 97 |
| *CitG* | triphosphoribosyl-dephospho-CoA synthase CitG | JHW33_RS02225 | WP_134706055.1 | WP_112151757.1 | 99 | WP_013574089.1 | 99 | WP_013574089.1 | 99 | WP_015695979.1 | 92 |
| *DcuS* | sensor histidine kinase DcuS | JHW33_RS24405 | WP_037032932.1 | WP_037032932.1 | 99 | WP_013578290.1 | 99 | WP_013578290.1 | 99 | WP_014341911.1 | 99 |
| *DcuR* | response regulator DcuR | JHW33_RS24410 | WP_200227982.1 | WP_014416701.1 | 99 | WP_014416701.1 | 99 | WP_013578289.1 | 99 | WP_014341910.1 | 97 |
| *GlrK* | sensor histidine kinase GlrK | JHW33_RS05205 | WP_200225233.1 | WP_112152427.1 | 99 | WP_015689470.1 | 99 | WP_041689061.1 | 99 | WP_037038457.1 | 98 |
| *GlrR* | two-component system response regulator GlrR | JHW33_RS21820 | WP_013574350.1 | WP_013574350.1 | 100 | WP_013574350.1 | 100 | WP_013574350.1 | 100 | WP_015696237.1 | 99 |
| *GlnL* | nitrogen regulation sensor histidine kinase GlnL | JHW33_RS16335 | WP_013577653.1 | WP_013577653.1 | 100 | WP_013577653.1 | 100 | WP_013577653.1 | 100 | WP_015699251.1 | 99 |
| *GlnG* | nitrogen regulation response regulator GlnG \| | JHW33_RS16340 | WP_013577654.1 | WP_013577654.1 | 100 | WP_013577654.1 | 100 | WP_013577654.1 | 100 | WP_015699252.1 | 99 |
| *BarA* | two-component sensor histidine kinase BarA | JHW33_RS19895 | WP_200224011.1 | WP_013573990.1 | 99 | WP_013573990.1 | 99 | WP_013573990.1 | 99 | WP_015695886.1 | 99 |
