# Supplementary material for: Comparative Genomics Assisted Functional Characterization of Rahnella aceris ZF458 as a Novel Plant Growth Promoting Rhizobacterium
Source: Front Microbiol. 2022 Apr 4;13:850084. doi: 10.3389/fmicb.2022.850084 (PMC9015054; doi:10.3389/fmicb.2022.850084)
Supplement: Supplementary file 15 [file Table_8.DOCX]

**Supplementary Table 8** Genes related to acid resistance in *R. aceris* ZF458 and other *Rahnella* strains.

| **Strain** |  | ***Rahnella aceris* ZF458** | | ***R. aquatilis* ZF7** | | ***R. aquatilis* HX2** | | ***Rahnella* sp. Y9602** | | ***R. aquatilis* ATCC 33071** | |
| --- | --- | --- | --- | --- | --- | --- | --- | --- | --- | --- | --- |
| **Genes** | **Product Definition** | **Locus Tag** | **Protein ID** | **Protein ID** | **Homology (%)** | **Protein ID** | **Homology (%)** | **Protein ID** | **Homology (%)** | **Protein ID** | **Homology (%)** |
| **Glutamate Decarboxylase system** | | | | | | | | | | | |
| *H-NS* | DNA-binding transcriptional regulator H-NS | JHW33_RS03955 | WP_013575950.1 | WP_013575950.1 | 100 | WP_013575950.1 | 100 | WP_013575950.1 | 100 | WP_013575950.1 | 100 |
| *RcsB* | transcriptional regulator RcsB | JHW33_RS00970 | WP_013574620.1 | WP_013574620.1 | 100 | WP_013574620.1 | 100 | WP_013574620.1 | 100 | WP_013574620.1 | 100 |
| **Lysine Decarboxylase system** | | | | | | | | | | | |
| *LdcC* | lysine decarboxylase LdcC | JHW33_21085 | WP_200224152.1 | WP_013574210.1 | 99 | WP_015689430.1 | 99 | WP_013574210.1 | 99 | WP_015696103.1 | 99 |
| **Ornithine Decarboxylase system** | | | | | | | | | | | |
| *SpeF* | ornithine decarboxylase SpeF | JHW33_RS12095 | WP_037033294.1 | WP_037033294.1 | 100 | WP_013576854.1 | 99 | WP_013576854.1 | 100 | WP_015698521.1 | 99 |
| **Adenine nucleoside deaminase** | | | | | | | | | | | |
| *add* | Adenosine deaminase | JHW33_RS05900 | WP_200225369.1 | WP_013575496.1 | 99 | WP_013575496.1 | 99 | WP_013575496.1 | 99 | WP_015697312.1 | 97 |
| **Aspartase** | | | | | | | | | | | |
| *aspA* | aspartate ammonia-lyase | JHW33_RS18640 | WP_200223781.1 | WP_013573737.1 | 99 | WP_013573737.1 | 99 | WP_013573737.1 | 99 | WP_014333637.1 | 99 |
| **Arginine deiminase** | | | | | | | | | | | |
| *arcA* | arginine deiminase | JHW33_RS13785 | WP_013577165.1 | WP_013577165.1 | 100 | WP_013577165.1 | 100 | WP_013577165.1 | 100 | WP_015698827.1 | 99 |
| **F1F0-ATPase** | | | | | | | | | | | |
| *atpE* | F0F1 ATP synthase subunit C | JHW33_RS16490 | WP_004093904.1 | WP_004093904.1 | 100 | WP_004093904.1 | 100 | WP_004093904.1 | 100 | WP_004093904.1 | 100 |
| *hemA* | glutamyl-tRNA reductase | JHW33_RS05445 | WP_013575588.1 | WP_013575588.1 | 100 | WP_013575588.1 | 100 | WP_013575588.1 | 100 | WP_015697396.1 | 99 |
| **Cyclopropane fatty acid synthesis** | | | | | | | | | | | |
| *cfa* | cyclopropane fatty acyl phospholipid synthase | JHW33_RS07900 | WP_013576089.1 | WP_013576089.1 | 100 | WP_013576089.1 | 100 | WP_013576089.1 | 100 | WP_015697798.1 | 99 |
| **Molecular chaperone** | | | | | | | | | | | |
| *dnak* | molecular chaperone DnaK | JHW33_RS13155 | WP_013577068.1 | WP_119262118.1 | 100 | WP_013577068.1 | 100 | WP_013577068.1 | 100 | WP_015698723.1 | 99 |
| *grpE* | nucleotide exchange factor GrpE | JHW33_RS11450 | WP_013576732.1 | WP_013576732.1 | 100 | WP_013576732.1 | 100 | WP_013576732.1 | 100 | WP_015698405.1 | 99 |
| *groL* | chaperonin GroEL | JHW33_RS18655 | WP_013573739.1 | WP_005120975.1 | 100 | WP_013573739.1 | 100 | WP_013573739.1 | 100 | WP_014333640.1 | 99 |
| *sspB* | ClpXP protease specificity-enhancing factor | JHW33_RS14120 | WP_013577236.1 | WP_013577236.1 | 100 | WP_013577236.1 | 100 | WP_013577236.1 | 100 | WP_015698879.1 | 97 |
| **Two Component Regulation System** | | | | | | | | | | | |
| *Rpos* | RNA polymerase sigma factor RpoS | JHW33_RS20035 | WP_200224020.1 | WP_013574019.1 | 100 | WP_013574019.1 | 99 | WP_013574019.1 | 99 | WP_013574019.1 | 99 |
| *EnvZ* | two-component system sensor histidine kinase EnvZ | JHW33_RS17865 | WP_013573600.1 | WP_013573600.1 | 100 | WP_013573600.1 | 100 | WP_013573600.1 | 100 | WP_014333528.1 | 99 |
| *OmpR* | two-component system response regulator OmpR | JHW33_RS17860 | WP_009635740.1 | WP_119261115.1 | 100 | WP_009635740.1 | 100 | WP_009635740.1 | 100 | WP_009635740.1 | 100 |
| *PmrA* | two-component system response regulator PmrA | JHW33_RS19015 | WP_013573810.1 | WP_013573810.1 | 100 | WP_013573810.1 | 100 | WP_013573810.1 | 100 | WP_014333693.1 | 98 |
| *PmrB* | two-component system sensor histidine kinase PmrB | JHW33_RS19010 | WP_200223845.1 | WP_112197543.1 | 100 | WP_013573809.1 | 99 | WP_013573809.1 | 99 | WP_014333692.1 | 91 |
| *phoP* | two-component system response regulator PhoP | JHW33_RS08835 | WP_013576218.1 | WP_013576218.1 | 100 | WP_013576218.1 | 100 | WP_013576218.1 | 100 | WP_013576218.1 | 100 |
| *phoQ* | two-component system sensor histidine kinase PhoQ | JHW33_RS08840 | WP_013576219.1 | WP_013576219.1 | 100 | WP_013576219.1 | 100 | WP_013576219.1 | 100 | WP_015697910.1 | 99 |
| **Small RNA** | | | | | | | | | | | |
| *RpoN* | RNA polymerase factor sigma-54 | JHW33_RS14225 | WP_013577258.1 | WP_013577258.1 | 100 | WP_013577258.1 | 100 | WP_013577258.1 | 100 | WP_015698898.1 | 99 |
| *hfq* | RNA chaperone Hfq | JHW33_RS18790 | WP_013573763.1 | WP_013573763.1 | 100 | WP_013573763.1 | 100 | WP_013573763.1 | 100 | WP_013573763.1 | 100 |
| **Other Regulation proteins** | | | | | | | | | | | |
| *hupA* | DNA-binding protein HU-alpha | JHW33_RS14950 | WP_013577396.1 | WP_013577396.1 | 100 | WP_013577396.1 | 100 | WP_013577396.1 | 100 | WP_013577396.1 | 100 |
| *dps* | DNA starvation/stationary phase protection protein Dps | JHW33_RS01310 | WP_015689551.1 | WP_015689551.1 | 100 | WP_015689551.1 | 100 | WP_013574696.1 | 99 | WP_015696542.1 | 99 |
| *crp* | cAMP-activated global transcriptional regulator  CRP | JHW33_RS18040 | WP_013573634.1 | WP_013573634.1 | 100 | WP_013573634.1 | 100 | WP_013573634.1 | 100 | WP_013573768.1 | 100 |
| *OmpC* | porin OmpC | JHW33_RS01005 | WP_013574627.1 | WP_013574627.1 | 100 | WP_015690049.1 | 100 | WP_015690049.1 | 100 | WP_015696493.1 | 99 |
| *OmpF* | porin | JHW33_RS04810 | WP_200227171.1 | WP_162906095.1 | 84 | WP_162906095.1 | 84 | WP_162906095.1 | 84 | WP_148267156.1 | 89 |
