# Supplementary material for: Comparative Genomics Assisted Functional Characterization of Rahnella aceris ZF458 as a Novel Plant Growth Promoting Rhizobacterium
Source: Front Microbiol. 2022 Apr 4;13:850084. doi: 10.3389/fmicb.2022.850084 (PMC9015054; doi:10.3389/fmicb.2022.850084)
Supplement: Supplementary file 16 [file Table_9.DOCX]

**Supplementary Table 9** Genes associated with flagella biosynthesis and chemotaxis in *R. aceris* ZF458 and other *Rahnella* strains.

| **Strain** |  | ***Rahnella aceris* ZF458** | | ***R. aquatilis* ZF7** | | ***R. aquatilis* HX2** | | ***Rahnella* sp. Y9602** | | ***R. aquatilis* ATCC 33071** | |
| --- | --- | --- | --- | --- | --- | --- | --- | --- | --- | --- | --- |
| **Genes** | **Product Definition** | **Locus Tag** | **Protein ID** | **Protein ID** | **Homology (%)** | **Protein ID** | **Homology (%)** | **Protein ID** | **Homology (%)** | **Protein ID** | **Homology (%)** |
| **Flagella biosynthesis genes** | | | | | | | | | | | |
| *flhD* | Flagellar transcriptional activator FlhD | JHW33_RS03120 | WP_013575090.1 | WP_013575090.1 | 100 | WP_013575090.1 | 100 | WP_013575090.1 | 100 | WP_015696908.1 | 99 |
| *flhC* | Flagellar transcriptional activator FlhC | JHW33_RS03125 | WP_013575091.1 | WP_013575091.1 | 100 | WP_013575091.1 | 100 | WP_013575091.1 | 100 | WP_015696909.1 | 99 |
| *motA* | Flagellar motor protein MotA | JHW33_RS03130 | WP_013575092.1 | WP_112151826.1 | 99 | WP_013575092.1 | 100 | WP_013575092.1 | 100 | WP_015696910.1 | 99 |
| *motB* | Flagellar motor protein MotB | JHW33_RS03135 | WP_013575093.1 | WP_013575093.1 | 100 | WP_013575093.1 | 100 | WP_013575093.1 | 100 | WP_015696911.1 | 96 |
| *flhB* | Flagellar biosynthesis protein FlhB | JHW33_RS03450 | WP_013575156.1 | WP_013575156.1 | 100 | WP_013575156.1 | 100 | WP_013575156.1 | 100 | WP_015696937.1 | 99 |
| *flhA* | Flagellar biosynthesis protein FlhA | JHW33_RS03455 | WP_037034835.1 | WP_015689760.1 | 100 | WP_015689760.1 | 99 | WP_013575157.1 | 99 | WP_015696938.1 | 98 |
| *flhE* | Flagellar protein FlhE | JHW33_RS03460 | WP_134706186.1 | WP_015689761.1 | 99 | WP_015689761.1 | 99 | WP_013575158.1 | 99 | WP_015696939.1 | 88 |
| *flgN* | FlgN family protein | JHW33_RS03465 | WP_013575160.1 | WP_119261478.1 | 99 | WP_015689765.1 | 99 | WP_013575160.1 | 100 | WP_015696941.1 | 91 |
| *flgM* | Anti-sigma28 factor FlgM | JHW33_RS03470 | WP_013575161.1 | WP_013575161.1 | 100 | WP_013575161.1 | 100 | WP_013575161.1 | 100 | WP_015696942.1 | 95 |
| *flgA* | Flagella basal body P-ring formation protein FlgA | JHW33_RS03475 | WP_013575162.1 | WP_013575162.1 | 100 | WP_013575162.1 | 100 | WP_013575162.1 | 100 | WP_148267122.1 | 93 |
| *flgB* | Flagellar basal body rod protein FlgB | JHW33_RS03480 | WP_013575163.1 | WP_013575163.1 | 100 | WP_013575163.1 | 100 | WP_013575163.1 | 100 | WP_015696944.1 | 99 |
| *flgC* | Flagellar basal body rod protein FlgC | JHW33_RS03485 | WP_013575164.1 | WP_013575164.1 | 100 | WP_013575164.1 | 100 | WP_013575164.1 | 100 | WP_013575164.1 | 100 |
| *flgD* | Flagellar basal body rod modification protein | JHW33_RS03490 | WP_013575165.1 | WP_013575165.1 | 100 | WP_013575165.1 | 100 | WP_013575165.1 | 100 | WP_015696945.1 | 97 |
| *flgE* | Flagellar hook protein FlgE | JHW33_RS03495 | WP_013575166.1 | WP_013575166.1 | 100 | WP_013575166.1 | 100 | WP_013575166.1 | 100 | WP_015696946.1 | 97 |
| *flgF* | Flagellar basal-body rod protein FlgF | JHW33_RS03500 | WP_013575167.1 | WP_013575167.1 | 100 | WP_013575167.1 | 100 | WP_013575167.1 | 100 | WP_015696947.1 | 99 |
| *flgG* | Flagellar basal body rod protein FlgG | JHW33_RS03505 | WP_013575168.1 | WP_013575168.1 | 100 | WP_013575168.1 | 100 | WP_013575168.1 | 100 | WP_015696948.1 | 99 |
| *flgH* | Flagellar basal body L-ring protein | JHW33_RS03510 | WP_071823641.1 | WP_071823641.1 | 100 | WP_071823641.1 | 100 | WP_071823641.1 | 100 | WP_072010213.1 | 98 |
| *flgI* | Flagellar basal body P-ring protein | JHW33_RS03515 | WP_013575170.1 | WP_013575170.1 | 100 | WP_013575170.1 | 100 | WP_013575170.1 | 100 | WP_015696950.1 | 99 |
| *flgJ* | Flagellar rod assembly protein/muramidase FlgJ | JHW33_RS03520 | WP_013575171.1 | WP_013575171.1 | 100 | WP_013575171.1 | 100 | WP_013575171.1 | 100 | WP_015696951.1 | 99 |
| *flgK* | Flagellar hook-associated protein FlgK | JHW33_RS03525 | WP_037034827.1 | WP_013575172.1 | 99 | WP_013575172.1 | 99 | WP_013575172.1 | 99 | WP_015696952.1 | 97 |
| *flgL* | Flagellar hook-associated protein FlgL | JHW33_RS03530 | WP_013575173.1 | WP_013575173.1 | 100 | WP_013575173.1 | 100 | WP_013575173.1 | 100 | WP_015696953.1 | 92 |
| *fliZ* | Flagella biosynthesis protein FliZ | JHW33_RS04635 | WP_013575776.1 | WP_013575776.1 | 100 | WP_013575776.1 | 100 | WP_013575776.1 | 100 | WP_015697538.1 | 98 |
| *fliA* | Flagellar biosynthesis sigma factor | JHW33_RS04630 | WP_013575777.1 | WP_013575777.1 | 100 | WP_013575777.1 | 100 | WP_013575777.1 | 99 | WP_015697539.1 | 98 |
| *fliC* | FliC/FljB family flagellin | JHW33_RS04625 | WP_013575778.1 | WP_013575778.1 | 100 | WP_013575778.1 | 100 | WP_013575778.1 | 100 | WP_015697540.1 | 49 |
| *fliD* | Flagellar capping protein | JHW33_RS04620 | WP_013575779.1 | WP_112152541.1 | 100 | WP_013575779.1 | 100 | WP_013575779.1 | 100 | WP_015697541.1 | 49 |
| *fliS* | Flagellar protein FliS | JHW33_RS04615 | WP_013575780.1 | WP_013575780.1 | 100 | WP_013575780.1 | 100 | WP_013575780.1 | 100 | WP_015697542.1 | 86 |
| *fliT* | Flagellar biosynthesis protein FliT | JHW33_RS04610 | WP_013575781.1 | WP_013575781.1 | 100 | WP_013575781.1 | 100 | WP_013575781.1 | 100 | WP_015697543.1 | 97 |
| *fliE* | Flagellar hook-basal body protein FliE | JHW33_RS04540 | WP_013575795.1 | WP_013575795.1 | 100 | WP_013575795.1 | 100 | WP_013575795.1 | 100 | WP_015697555.1 | 98 |
| *fliF* | Flagellar M-ring protein FliF | JHW33_RS04535 | WP_015690044.1 | WP_013575796.1 | 100 | WP_015690044.1 | 100 | WP_013575796.1 | 99 | WP_015697556.1 | 99 |
| *fliG* | Flagellar motor switch protein FliG | JHW33_RS04530 | WP_013575797.1 | WP_013575797.1 | 100 | WP_013575797.1 | 100 | WP_013575797.1 | 100 | WP_015697557.1 | 99 |
| *fliH* | Flagellar assembly protein FliH/type III secretion system HrpE | JHW33_RS04525 | WP_187514434.1 | WP_187514434.1 | 100 | WP_173362102.1 | 99 | WP_173362102.1 | 99 | WP_193785518.1 | 96 |
| *fliI* | Flagellum-specific ATP synthase | JHW33_RS04520 | WP_013575799.1 | WP_013575799.1 | 100 | WP_013575799.1 | 100 | WP_013575799.1 | 100 | WP_015697559.1 | 99 |
| *fliJ* | Flagellar biosynthesis chaperone | JHW33_RS04515 | WP_013575800.1 | WP_013575800.1 | 100 | WP_013575800.1 | 100 | WP_013575800.1 | 100 | WP_015697560.1 | 97 |
| *fliK* | Flagellar hook-length control protein-like protein | JHW33_RS04510 | WP_200225187.1 | WP_015690045.1 | 99 | WP_015690045.1 | 99 | WP_013575801.1 | 99 | WP_015697561.1 | 87 |
| *fliL* | Flagellar basal body-associated protein FliL | JHW33_RS04505 | WP_037034434.1 | WP_013575802.1 | 99 | WP_013575802.1 | 99 | WP_013575802.1 | 100 | WP_015697562.1 | 94 |
| *fliM* | Flagellar motor switch protein FliM | JHW33_RS04500 | WP_013575803.1 | WP_013575803.1 | 100 | WP_013575803.1 | 100 | WP_013575803.1 | 100 | WP_015697563.1 | 98 |
| *fliN* | Flagellar motor switch protein FliN | JHW33_RS04495 | WP_013575804.1 | WP_013575804.1 | 100 | WP_013575804.1 | 100 | WP_013575804.1 | 100 | WP_013575804.1 | 100 |
| *fliO* | Flagellar biosynthetic protein FliO | JHW33_RS04490 | WP_200225186.1 | WP_119261720.1 | 99 | WP_013575805.1 | 99 | WP_013575805.1 | 99 | WP_015697564.1 | 97 |
| *fliP* | Flagellar biosynthesis protein FliP | JHW33_RS04485 | WP_037034611.1 | WP_037034611.1 | 100 | WP_037034611.1 | 99 | WP_037034611.1 | 99 | WP_037039578.1 | 98 |
| *fliQ* | Flagellar biosynthetic protein FliQ | JHW33_RS04480 | WP_013575807.1 | WP_013575807.1 | 100 | WP_013575807.1 | 100 | WP_013575807.1 | 100 | WP_015697566.1 | 99 |
| *fliR* | Flagellar biosynthesis protein FliR | JHW33_RS04475 | WP_013575808.1 | WP_013575808.1 | 100 | WP_013575808.1 | 100 | WP_013575808.1 | 100 | WP_015697567.1 | 99 |
| **Chemotaxis genes** | | | | | | | | | | | |
| *cheA* | Chemotaxis protein CheA | JHW33_RS03140 | WP_153375938.1 | WP_153375938.1 | 100 | WP_153375938.1 | 100 | WP_153375938.1 | 100 | WP_193785491.1 | 98 |
| *cheW* | Purine-binding chemotaxis protein | JHW33_RS03145 | WP_013575095.1 | WP_013575095.1 | 100 | WP_013575095.1 | 100 | WP_013575095.1 | 100 | WP_013575095.1 | 100 |
| *mcp* | methyl-accepting chemotaxis protein | JHW33_RS16595 | WP_013577700.1 | WP_013577700.1 | 100 | WP_015689458.1 | 100 | WP_013577700.1 | 100 | WP_015696208.1 | 98 |
| *tar* | methyl-accepting chemotaxis protein | JHW33_RS16830 | WP_037033855.1 | WP_112151993.1 | 99 | WP_013573392.1 | 99 | WP_013573774.1 | 99 | WP_015699295.1 | 97 |
| *cheR* | Chemotaxis methyltransferase CheR | JHW33_RS03430 | WP_013575152.1 | WP_013575152.1 | 100 | WP_013575152.1 | 100 | WP_013575152.1 | 100 | WP_015696934.1 | 99 |
| *cheB* | chemotaxis response regulator protein-glutamate methylesterase | JHW33_RS03435 | WP_013575153.1 | WP_013575153.1 | 100 | WP_013575153.1 | 100 | WP_013575153.1 | 100 | WP_015696935.1 | 99 |
| *cheY* | chemotaxis response regulator CheY | JHW33_RS03440 | WP_013575154.1 | WP_013575154.1 | 100 | WP_013575154.1 | 100 | WP_013575154.1 | 100 | WP_013575154.1 | 100 |
| *cheZ* | Chemotaxis regulator CheZ | JHW33_RS03445 | WP_013575155.1 | WP_013575155.1 | 100 | WP_013575155.1 | 100 | WP_013575155.1 | 100 | WP_015696936.1 | 99 |
