# Supplementary material for: Comparative Genomics Assisted Functional Characterization of Rahnella aceris ZF458 as a Novel Plant Growth Promoting Rhizobacterium
Source: Front Microbiol. 2022 Apr 4;13:850084. doi: 10.3389/fmicb.2022.850084 (PMC9015054; doi:10.3389/fmicb.2022.850084)
Supplement: Supplementary file 17 [file Table_10.DOCX]

**Supplementary Table 10** Genes related to quorum sensing in *R. aceris* ZF458 and other *Rahnella* strains.

| **Strain** |  | ***Rahnella aceris* ZF458** | | ***R. aquatilis* ZF7** | | ***R. aquatilis* HX2** | | ***Rahnella* sp. Y9602** | | ***R. aquatilis* ATCC 33071** | |
| --- | --- | --- | --- | --- | --- | --- | --- | --- | --- | --- | --- |
| **Genes** | **Product Definition** | **Locus Tag** | **Protein ID** | **Protein ID** | **Homology (%)** | **Protein ID** | **Homology (%)** | **Protein ID** | **Homology (%)** | **Protein ID** | **Homology (%)** |
| *luxS* | S-ribosylhomocysteine lyase | JHW33_RS20115 | WP_037034141.1 | WP_013574031.1 | 99 | WP_013574031.1 | 99 | WP_013574031.1 | 99 | WP_015695926.1 | 99 |
| *lsrG* | (4S)-4-hydroxy-5-phosphonooxypentane-2,3-dione isomerase | JHW33_RS04280 | WP_037034453.1 | WP_037034453.1 | 100 | WP_013575888.1 | 98 | WP_013575888.1 | 98 | WP_015697597.1 | 97 |
| *lsrF* | 3-hydroxy-5-phosphonooxypentane-2,4-dione thiolase | JHW33_RS04275 | WP_013575889.1 | WP_013575889.1 | 100 | WP_013575889.1 | 100 | WP_013575889.1 | 100 | WP_015697598.1 | 99 |
| *lsrB* | autoinducer 2 ABC transporter substrate-binding protein LsrB | JHW33_RS04270 | WP_013575890.1 | WP_013575890.1 | 100 | WP_013575890.1 | 100 | WP_013575890.1 | 100 | WP_015697599.1 | 98 |
| *lsrD* | autoinducer 2 ABC transporter permease LsrD | JHW33_RS04265 | WP_134706854.1 | WP_119261737.1 | 99 | WP_013575891.1 | 99 | WP_013575891.1 | 99 | WP_015697600.1 | 95 |
| *lsrC* | autoinducer 2 ABC transporter permease LsrC | JHW33_RS04260 | WP_200225176.1 | WP_119261738.1 | 99 | WP_015690055.1 | 99 | WP_013575892.1 | 99 | WP_015697601.1 | 94 |
| *lsrA* | autoinducer 2 ABC transporter ATP-binding protein LsrA | JHW33_RS04255 | WP_200225175.1 | WP_119261739.1 | 99 | WP_015690056.1 | 99 | WP_013575893.1 | 99 | WP_015697602.1 | 94 |
| *lsrR* | transcriptional regulator LsrR | JHW33_RS04250 | WP_013575894.1 | WP_013575894.1 | 100 | WP_013575894.1 | 100 | WP_013575894.1 | 100 | WP_015697603.1 | 98 |
| *lsrK* | autoinducer-2 kinase | JHW33_RS04245 | WP_200227166.1 | WP_013575895.1 | 99 | WP_013575895.1 | 99 | WP_013575895.1 | 99 | WP_015697604.1 | 97 |
| *ydiK* | AI-2E family transporter YdiK | JHW33_RS07985 | WP_013576106.1 | WP_013576106.1 | 100 | WP_013576106.1 | 100 | WP_013576106.1 | 100 | WP_015697811.1 | 95 |
| *tqsA* | AI-2E family transporter | JHW33_RS09035 | WP_037037317.1 | WP_119261816.1 | 99 | WP_013576262.1 | 99 | WP_013576262.1 | 99 | WP_015697945.1 | 95 |
| *perM* | AI-2E family transporter | JHW33_RS10570 | WP_013576556.1 | WP_013576556.1 | 100 | WP_013576556.1 | 100 | WP_013576556.1 | 100 | WP_015698250.1 | 99 |
